# Field evaluation of ascorbic acid seed priming on growth, physiology, yield, and nutritional quality of broccoli (*Brassica oleracea L.* var. *italica ‘Gypsy’*)

**DOI:** 10.3389/fpls.2026.1897446

**Published:** 2026-09-16

**Authors:** Vijaya R. Mohan, Andrew M. Hammermeister, Lord Abbey, Mason T. Macdonald

**Affiliations:** Department of Plant, Food, and Environmental Sciences, Dalhousie University, Truro, NS, Canada

**Keywords:** ascorbic acid, *Brassica oleracea*, nutrient composition, photosynthetic pigments, seed priming, sustainable agriculture, yield components

## Abstract

Seed priming with ascorbic acid (AsA) has been widely recognized for enhancing early plant development; however, its effectiveness in improving growth, physiology, yield, and nutritional quality of broccoli under field conditions remains limited. This two-year field study evaluated the effects of AsA seed priming on broccoli under Atlantic Canada soil conditions using a randomized complete block design across two field sites. Four treatments were assessed: non-primed control, water-primed (0 mgL^-1^), and AsA-primed seeds at 1 mgL^-1^ and 10 mgL^-1^. AsA priming significantly (p < 0.05) enhanced plant height, canopy length, stem girth, and leaf area, with the greatest improvements observed at 10 mgL^-1^, resulting in increases of 17.4%, 19.7%, 51.7%, and 69.4%, respectively, compared to the control. Net photosynthesis, stomatal conductance, transpiration, and chlorophyll index, were also significantly improved (p < 0.001). Biochemical analysis showed significant increases (p < 0.05) in chlorophyll *a* and carotenoid content at 10 mgL^-1^. Meanwhile phenolics, flavonoids, and most nutrients were not significantly affected (p ≥ 0.05), except for a slight but significant (p < 0.05) reduction in phosphorus concentration at 10 mgL^-1^. Yield components were significantly (p < 0.05) improved by AsA priming, with 10 mgL^-1^ increasing primary yield by 38.4% and secondary yield by 80.4%, whereas 1 mgL^-1^ significantly (p < 0.05) increased the number of secondary heads per plant by 15.3% compared with the control. Overall, AsA seed priming enhanced growth, physiological performance, and yield without compromising nutritional quality, demonstrating its potential as a practical and sustainable approach to improve broccoli production under field conditions.

## 1 Introduction

*Brassica oleracea L*. var. *italica* is a member of the Brassicaceae family and is commonly known as broccoli. The Brassicaceae family comprises 372 genera and 4372 species, and most of them are of great economic importance (Catalogue of Life, 2026). Among *Brassica* crops, broccoli has attracted increasing attention in recent years because of its exceptional nutritional profile and associated health benefits. It is a rich source of antioxidants, vitamins C and E, glucosinolates, carotenoids, polyphenols, and essential minerals. These bioactive compounds contribute to strengthening the human immune system against acute infections and play an important role in reducing the risk of cancer and cardiovascular diseases (Syed et al., 2023; Aires, 2015; Fahey, 2015; Andrés et al., 2025; Mohan et al., 2025). Due to Canada’s short growing season and geographic limitations, the country depends heavily on imported vegetables, including broccoli, to meet year-round demand. In 2024, Canada imported 154,830 metric tons of cauliflower and headed broccoli (the combined trade category under which broccoli is reported), valued at CAD 346.3 million and representing 7.9% of the total value of Canadian field vegetable imports which is third-largest imported field vegetable category after lettuce and onions (AAFC, 2024). This trend toward greater import dependence highlights the need to improve broccoli production, not only in Canada but around the world, to strengthen food security.

Modern agriculture depends heavily on chemical pesticides, and synthetic fertilizers for crop production (Gamage et al., 2023). However, their excessive use poses health risks and can pollute soil, water, and air while affecting non-target organisms and promoting pesticide resistance. Organic farming offers a more sustainable alternative by improving soil health and reducing environmental impacts (Finez and Talimbay, 2023; Zhou et al., 2025). Combining organic practices with modern technologies can further enhance productivity and sustainability, while also supporting long-term food security (Gamage et al., 2023). To address these challenges, environmentally friendly approaches that enhance crop performance without increasing chemical inputs are gaining attention, among which seed priming has emerged as a promising strategy.

Seed priming is a widely used agricultural technique that enhances seed germination, seedling growth, stress tolerance, and overall plant biomass (Hussain et al., 2017; Paparella et al., 2015; MacDonald and Mohan, 2025). Priming involves incubating seeds with a seed priming agent (SPA) to improve seedling development (MacDonald et al., 2009). Various compounds have proven effective as SPAs, including salicylic acid, acetylsalicylic acid, and glycine betaine, which improve carrot germination (Lada et al., 2005), as well as 5-hydroxybenzimidazole and pyroligneous acid, which enhance yield and stress resistance in tomato and rice, respectively (MacDonald et al., 2010; Simma et al., 2017). Naturally occurring antioxidants such as lycopene, β-carotene, and AsA have also been shown to increase tomato dry mass and photosynthetic performance (MacDonald et al., 2009). Among seed priming agents, AsA has received considerable attention due to its low cost, accessibility, and proven effectiveness across diverse crops. Ascorbic acid (AsA) is also permitted for use in organic agriculture, further supporting its suitability for sustainable crop production (Government of Canada, 2021), AsA which is commonly known as Vitamin C, is a water-soluble antioxidant widely present in plant tissues, occurring in multiple cellular compartments, including chloroplasts and cell walls, where it plays a central role in protecting plants against oxidative stress (Akram et al., 2017). It functions as an enzyme cofactor involved in photosynthesis, hormone biosynthesis (ethylene and gibberellins), anthocyanin production, cell division, growth regulation, and signal transduction (Smirnoff and Wheeler, 2000; Gallie, 2013). Numerous studies demonstrate that AsA seed priming enhances plant performance by improving germination, biomass accumulation, photosynthetic efficiency, and stress tolerance. For example, under drought-stress conditions, tomato seeds treated with 1 mg L^−1^ and 10 mg L^−1^ AsA exhibited a 40% increase in shoot dry mass, a 50% increase in leaf area, and a 223% rise in net photosynthetic rate compared with untreated, drought-stressed controls (MacDonald et al., 2009). Similarly, wheat seeds primed with 50 mgL^-1^ AsA produced approximately 26% higher grain yield, along with moderate improvements in germination (T. Shah et al., 2019). In rice, AsA priming at 10 mgL^-1^ and 20 mgL^-1^ increased germination rates and dry mass accumulation (Anwar et al., 2013). Research shows that AsA priming at 10 mg L^−1^ significantly enhanced germination index, shoot biomass, photosynthetic rates, root development, and drought tolerance in ‘Gypsy’ broccoli seedlings subjected to imposed drought stress under greenhouse conditions (Cai et al., 2024; MacDonald et al., 2022; Mohan et al., 2026). In sunflower, the combined application of 200mgL^-1^ salicylic acid and 100 mgL^-1^ AsA resulted in the highest grain yield (2.72 t ha^−1^) and oil content (52%) (Davodi et al., 2019). Although research in broccoli and quinoa has primarily focused on early growth stages, AsA priming significantly increased biomass accumulation in both species. In broccoli, 10 mg L^−1^ AsA priming increased shoot dry biomass by 38% in regularly watered seedlings, with even larger relative gains up to 92% under drought stress (Cai et al., 2024), while in quinoa, AsA priming improved biomass by up to 49% under saline conditions (Shah et al., 2025).

Despite the growing evidence supporting the benefits of AsA priming in several crops, most studies have been conducted under controlled or early growth conditions. However, a limited number of field-based studies in crops such as wheat, sunflower and rice have demonstrated that AsA priming can enhance growth, yield, and stress tolerance under real agricultural conditions (Zhang et al., 2025; Shah et al., 2019; Davodi et al., 2019). However, there is limited research on the impact of AsA seed priming in promoting the growth and development of broccoli under field conditions. This field evaluation study not only extends the assessment of AsA priming beyond controlled seedling conditions but also permits the measurement of agronomically and nutritionally relevant outcomes, including marketable primary and secondary head yield, as well as the biochemical and nutritional quality of harvested heads. Evaluating plants without experimentally imposed stress further enables the direct effects of AsA priming on crop performance to be distinguished from those associated with stress mitigation. However, it remains unknown whether benefits observed at the seedling stage persist throughout the crop cycle and translate into improved yield and quality at harvest, and whether these responses depend on the presence of stress in broccoli. This study addresses this knowledge gap by evaluating the effects of AsA on plant growth, development and yield of broccoli. In this study, it was hypothesized that seed priming with AsA enhances germination performance, plant growth, physiological functioning, and biomass accumulation in broccoli. Furthermore, it was hypothesized that optimal AsA concentrations would stimulate plant growth as well as physiological and biochemical activities, thereby translating early developmental advantages into improved yield performance, including both yield quantity and quality under field conditions. Overall, AsA seed priming is expected to provide a sustainable strategy to enhance broccoli productivity without increasing chemical inputs. This study provides insight into the role of AsA seed priming as a practical tool to enhance broccoli growth and productivity, supporting reduced chemical dependency, improved crop performance, and strengthened food security both in Canada and globally.

## 2 Materials and methods

### 2.1 Experimental design and AsA treatment

This experiment was conducted as a two-year field study to evaluate the effects of AsA seed priming on broccoli production. A randomized complete block design (RCBD) was employed, with two different field sites and two field seasons used as blocking factors. The experiment consisted of four seed-priming treatments: non-primed seeds (control), seeds primed with water only (0 mg/L), seeds primed with 1 mg/L AsA, and seeds primed with 10 mg/L AsA. Control seeds were not exposed to any priming solution; they were placed dry in an empty 250 mL flask and subjected to the same shaker incubation (150 rpm, 20 °C, 24 h, darkness) as the primed treatments, then handled identically thereafter. The broccoli cultivar ‘Gypsy’ was selected for this study because it is widely cultivated across North America, particularly in Canada, and represents an important commercial variety within regional production systems. In addition, ‘Gypsy’ has been consistently used in previous AsA seed priming studies conducted (Cai et al., 2024; MacDonald et al., 2022), enabling direct comparison of the present field results with prior greenhouse findings.

### 2.2 AsA seed priming

A 1000 mgL^-1^ stock solution of AsA, with a purity of 99%, in reagent-grade crystalline form from Sigma-Aldrich, Oakville, ON, Canada, was prepared using deionized water. This stock solution was diluted to concentrations of 1 mgL^-1^ and 10 mgL^-1^ with deionized water. All AsA solutions were freshly prepared immediately prior to use, and incubation was conducted in darkness to prevent photooxidation. The pH of the AsA treatment solutions decreased slightly with increasing concentration and was not adjusted: water primed (6.50), 1 mgL^-1^ (6.41) and 10 mgL^-1^ (6.18) reflecting the inherent acidity of ascorbic acid in aqueous solution. Each of the four priming treatments was transferred to separate 250 mL flasks. There was one additional flask with pure deionized water to represent water priming (0 mgL^-1^). Exactly 100 seeds were added to each flask containing 200 mL of AsA solution and placed in a G24 Environmental Incubator Shaker (NB Scientific Co., Inc., Woodbridge Township, NJ, USA) set at 150 rpm and 20 °C. After 24 h of incubation in an environmental incubator shaker, the contents of each flask were poured through a sieve, and the seeds were gently dried using filter paper. The seeds were then sown immediately in seed trays containing Pro-Mix. Seeds were not surface sterilized prior to priming. Commercially certified seeds were used, and the priming conditions (24 h at 20 °C in darkness in either deionized water or AsA solution) provided limited opportunity for microbial proliferation. Following priming, seeds were sown immediately after surface drying, and no seed decay, damping-off, or other symptoms of seedling disease were observed in either experimental year. This procedure is consistent with previously reported AsA seed-priming protocols in broccoli. This seed priming procedure was adapted from and conducted following the method described by (Cai et al., 2024). Control seeds did not undergo any priming treatment.

### 2.3 Greenhouse growing conditions

Seeds were sown in a greenhouse in May 2024 and July 2025 using two 36-cell seed trays (Feeds N Needs, Truro, Canada), each tray measuring 12.7 × 8.5 × 5.7 cm. In 2025, the initial sowing conducted in May was lost due to an extreme heat event that coincided with a malfunction of the greenhouse ventilation system, resulting in seedling mortality across all treatments. Consequently, the experiment was re-established in July 2025 using freshly sown seeds for all treatment groups. To account for potential variation associated with differences in growing conditions between experimental seasons, year (season) was included as a blocking factor in the statistical analysis. Each cell was filled with approximately 7.5 grams of Pro-Mix Plain BX growing medium (Premier Tech Ltd, QC, Canada). The greenhouse provided optimal growing conditions for the seedlings, with daily and night temperatures ranging between 25 °C and 18 °C and approximately 12 hours of natural light. Broccoli seeds for this study were purchased from Halifax Seed, Halifax, NS, Canada. The potting medium, Pro-Mix BX, has a pH range of 5.5–6.5, electrical conductivity of 1.3–2.0 mmhos cm^-1^, air porosity of 17–22% by volume, and water holding capacity of 700–900% by weight. This pH range falls within the optimal range for broccoli seedling production; therefore, no pH adjustment was needed. After six weeks of greenhouse growth, seedlings were transplanted into the field for further evaluation under field conditions.

### 2.4 Site description

The field study was conducted during the 2024 and 2025 growing seasons at two sites located at the Dalhousie Agriculture campus in Bible Hill, Nova Scotia, Canada, namely, namely the Site A (45°23′33″ N, 63°15′48″ W) and the Site B (45°22′29″ N, 63°15′47″ W). The soil at both locations is a well-drained Pugwash series loam, classified as an Orthic Humo-Ferric Podzol according to the Canadian System of Soil Classification (Anderson and Smith, 2011), and characterized as a well-drained Orthic Humo-Ferric Podzol. Total precipitation from May to November was 732.5 mm in 2024 and 459.4 mm in 2025 (**Figure 1**). The average air temperature during the growing season was 14.1 °C in 2024 and 14.0 °C in 2025 (**Figure 2**). The previous crop grown at experimental sites during the 2023 growing season consisted of an oats, pea, and vetch cover crop mixture.

**Figure 1:**
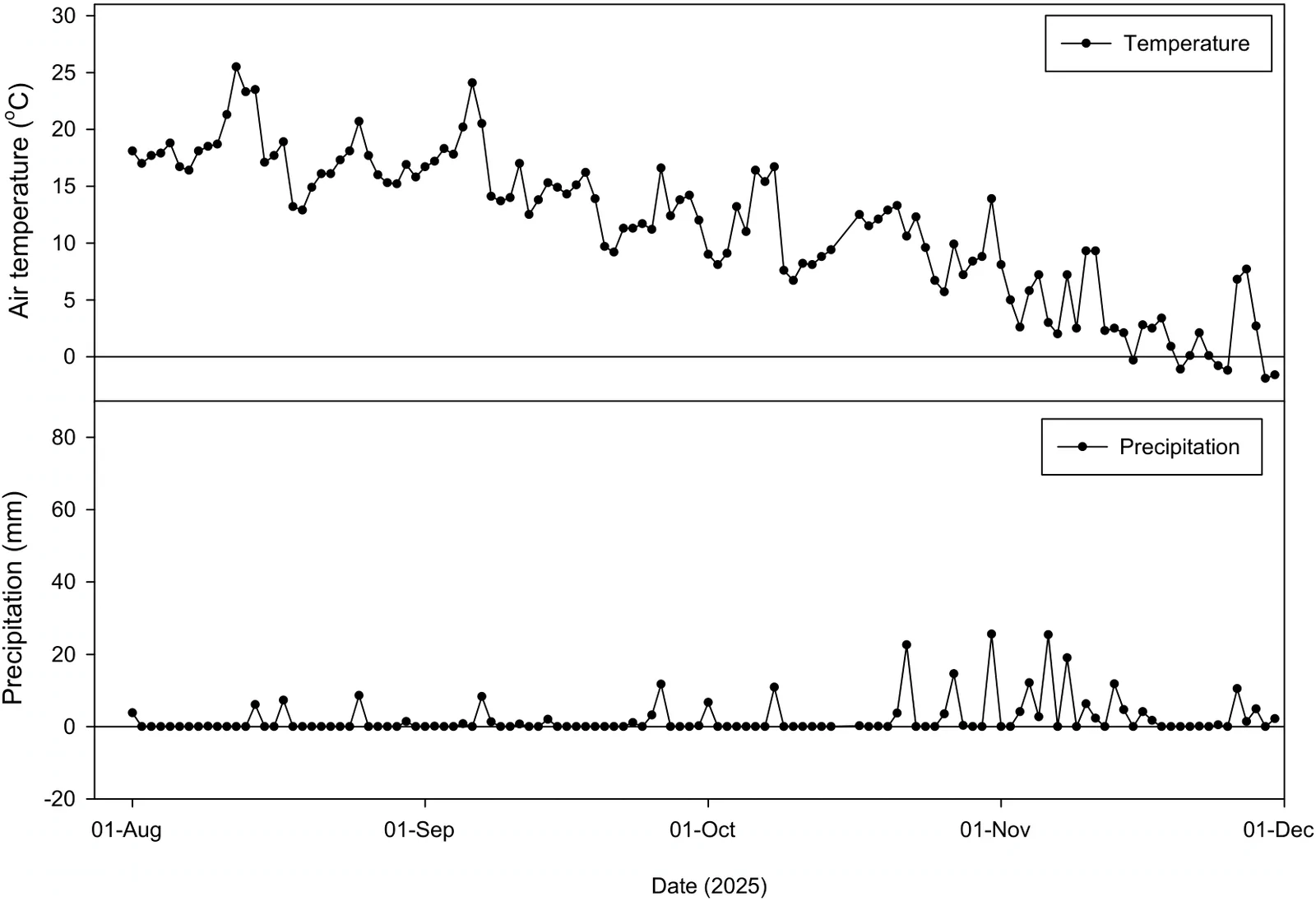
Air temperature and precipitation recorded during the field experimental period in 2025. Weather data were obtained from the local weather station located in Debert, Nova Scotia, Canada, and sourced from the Government of Canada meteorological database. Data points were used to generate line graphs, and the complete dataset is provided in the **Supplementary Materials**.

**Figure 2:**
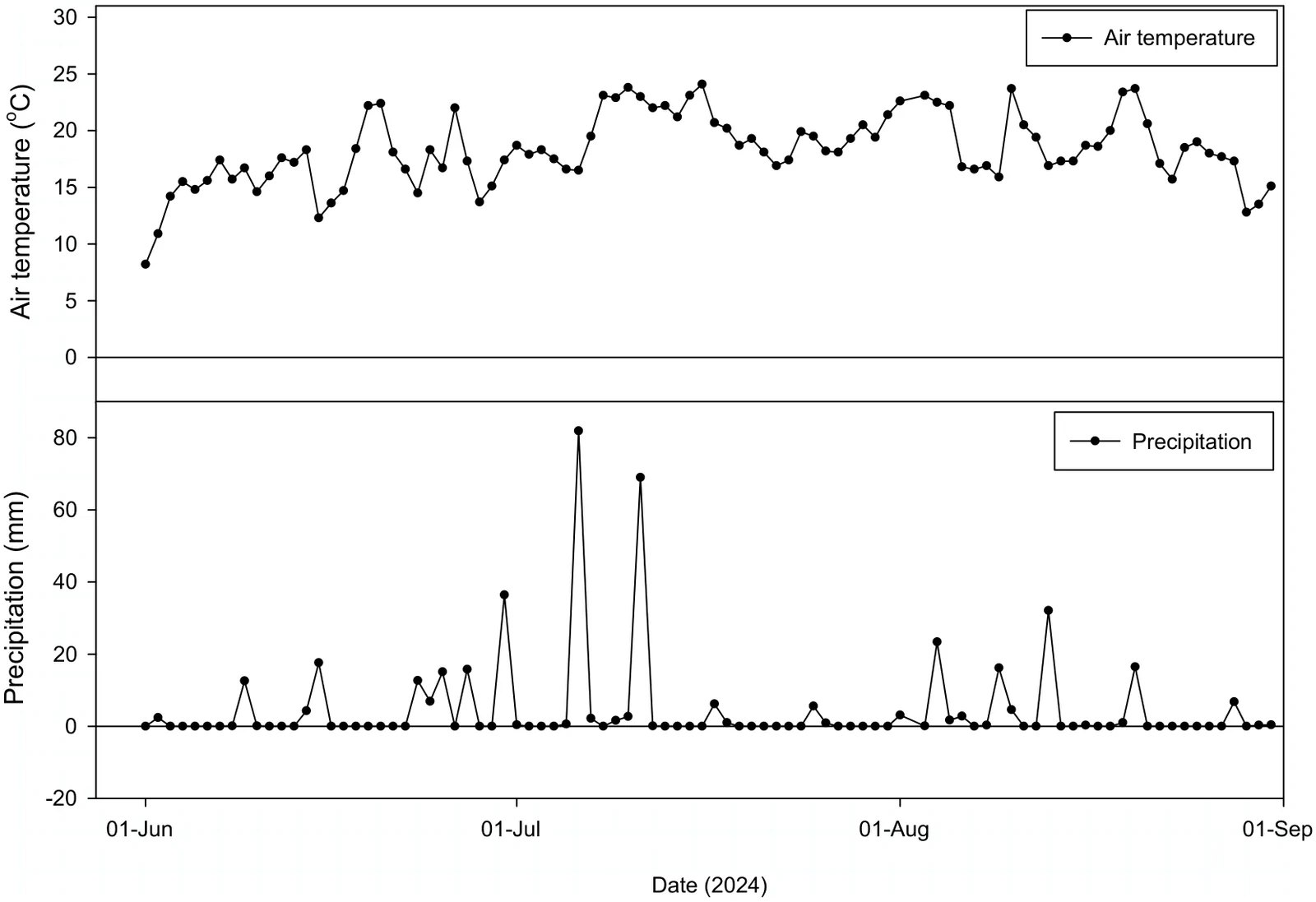
Mean average daily air temperature and precipitation recorded during the field experimental period in 2024. Weather data were obtained from the local weather station located in Debert, Nova Scotia, Canada, and sourced from the Government of Canada meteorological database. Data points were used to generate line graphs, and the complete dataset is provided in the **Supplementary Materials**.

**Figure 3:**
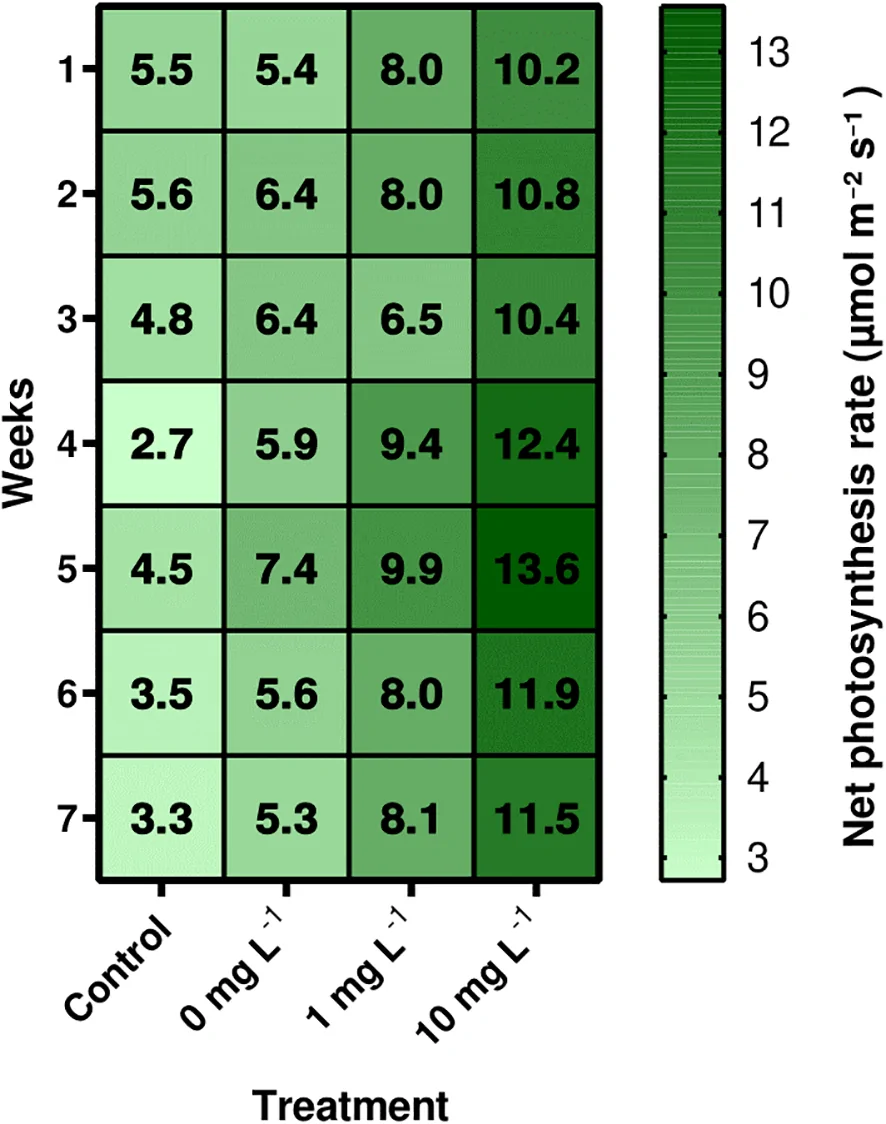
Heat map illustrating the interaction effects of sampling weeks and treatments on net photosynthesis rate (µmol m^−2^s^−1^). Color intensity represents the magnitude of net photosynthesis. Statistical significance was evaluated using Fisher’s least significant difference (LSD) test at p < 0.05.

### 2.5 Field configuration and management

The experiment was arranged in a randomized complete block design (RCBD) in which each combination of site (A or B) and year (2024 or 2025) was treated as a block, giving four blocks in total. Within each block, the four AsA treatments were each replicated in five plots (4 treatments × 5 replicate plots = 20 plots per site per year; 40 plots per year and 80 plots across the two seasons), with each plot measuring 1 m × 2 m. Each plot contained nine plants with same treatment, spaced 30 cm apart with a 1 m buffer zone between plots to minimize treatment interference. Seedlings were randomly allocated to plots within each block to ensure an even distribution of treatments across experimental units. Each plot was considered the experimental unit for all statistical analyses. Accordingly, each plot, consisting of nine plants, was treated as a single replicate.

Following transplanting, plants were maintained under field conditions throughout the growing season. Irrigation was applied as required using sprinkler systems to prevent visible moisture stress and to maintain soil moisture within an optimal range below field capacity, based on regular field observations and prevailing weather conditions. Irrigation frequency and duration varied depending on weather conditions; typically, water was applied 2–3 times per week for approximately 1–2 hours per event during dry periods. The Demonstration Garden system delivered approximately 4–5 mm h^−1^, while the Chef’s Garden system delivered approximately 6 mm h^−1^.One week prior to transplanting, feather meal fertilizer (N–P–K: 13–0–0) was applied at a rate of 100 kg ha^−1^ at each field site. The feather meal was sourced from Gaia Green Organics (Surrey, BC, Canada). Feather meals were selected as the organic nitrogen source. Pre-transplant soil analyses (**Supplementary Data 3**, **4**) indicated adequate plant-available concentrations of phosphorus, potassium, and micronutrients at both study sites; therefore, only nitrogen supplementation was required. Weed management was carried out manually throughout the experiment to minimize plant competition. Pest control was achieved through the application of a biological insecticide, *Dipel^®^ 2XDF*, at a rate of 400 g ha^−1^ every 10 days (Nufarm, Calgary, AB, Canada). The broccoli plants were grown under field conditions for an additional eight weeks prior to the commencement of harvesting.

### 2.6 Data collection

#### 2.6.1 Soil sampling and site characterization

Field soil samples were collected from a depth of 0–20 cm at five randomly selected locations within each site, and soil samples from each site were combined to form a composite sample. The composite soil samples were submitted to the Nova Scotia Department of Agriculture Laboratory Services (Truro, NS, Canada) for chemical nutrient analysis using the Mehlich III extraction method (Mehlich, 1984). This method was used to determine plant-available nutrient concentrations, which were quantified using an inductively coupled plasma optical emission spectrometer (ICP-OES 725, Agilent Technologies, California, USA). Soil analysis was conducted prior to transplanting to ensure adequate nutrient availability for crop establishment. Detailed soil analysis reports are provided in **Supplementary Data**.

#### 2.6.2 Measuring growth and yield response

Throughout the field growing period, weekly measurements were recorded for several plant growth parameters, including plant height, stem girth, canopy length, and leaf area. Measurements were taken using a caliper, meter stick, and measuring tape. For leaf assessments, the third fully expanded leaf was selected from randomly chosen plants within each plot (n = 9 plants per plot). Leaf and gas exchange measurements were consistently conducted on this leaf position throughout the duration of the experiment. Chlorophyll index was measured using a Minolta SPAD-504 meter (Konica Minolta, Ramsey, NJ, USA). Pn, E, and Gs were measured using an LCi T Gas Exchange System (ADC Bioscientific, Hoddesdon, UK). Each gas exchange measurement was recorded three times at intervals of 30, 60, and 90 seconds, and the reported Pn, E, and Gs values represent the average of the three readings to improve measurement reliability and minimize variability associated with instantaneous fluctuations in photosynthetic activity. Physiological data for the 2025 growing season were unavailable for the first four sampling weeks due to a temporary equipment failure. Data collection resumed thereafter, and all subsequent measurements were conducted according to the experimental schedule.

Harvesting began eight weeks after transplanting. For the primary harvest, all broccoli heads were collected on the same day to ensure consistency in data collection. Approximately two to three weeks later, secondary heads were harvested following a decline in ambient temperatures, allowing assessment of re-growth and additional yield under cooler conditions. In the 2024 growing season, the primary harvest was conducted on August 19, followed by the secondary harvest on September 6. In the 2025 growing season, the primary harvest occurred on October 7, and the secondary harvest was completed on October 24. Following completion of the secondary harvest each year, the experiment was concluded and post harvest data analysis was initiated.

Harvested heads were immediately transported to the laboratory, cleaned, and measured. Data collected included head counts per treatment, fresh weight determined using an analytical balance, and dry weight measurements following drying. Head weights were converted to yield by calculating biomass on a grams-per-plant basis. Plant tissue samples were then submitted to the Nova Scotia Department of Agriculture Laboratory Services (Truro, NS, Canada) for chemical analysis. Tissue nutrient analysis included nitrogen (N), phosphorus (P), potassium (K), calcium (Ca), magnesium (Mg), iron (Fe), manganese (Mn), copper (Cu), zinc (Zn), boron (B), and sodium (Na). Harvest data, together with weekly growth measurements, were subsequently used for statistical analysis of treatment effects.

### 2.7 Measuring biochemical response

Harvested head samples from each plant within each treatment at harvest were flash-frozen in liquid nitrogen and then ground into a fine powder using a pre-cooled mortar and pestle. The powdered samples were immediately stored at -80 °C for subsequent analyses.

#### 2.7.1 Chlorophyll and carotenoids

Total chlorophyll a, chlorophyll b, and carotenoid contents were determined following the method of (Lichtenthaler, 1987). A 0.2 g ground sample was placed into a sterile 50 mL Falcon tube, and 10 mL of 80% acetone *(v/v)* was added. The mixture was vortexed for 1 min and then centrifuged at 12,000 g for 15 min. Subsequently, 1 mL of the supernatant was transferred into a cuvette, and absorbance readings were taken at 646.8 nm (A_646.8_) and 663.2 nm (A_663.2_) using a UV-Vis spectrophotometer (Tecan Infinite^®^ M200 PRO, Morrisville, NC, USA), with 80% acetone serving as the blank. Chlorophyll a and chlorophyll b concentrations were then calculated using the (Equations 1, 2) below. For the carotenoid concentration, the absorbance was measured at 470 nm and calculated using (Equation 3). The calculated pigment concentrations (μg mL^−1^) were converted to a fresh-weight basis (μg g^−1^ FW) using the equation (C × V)/W, where C represents the pigment concentration calculated from the respective formula (μg mL^−1^), V is the total extract volume (10 mL), and W is the fresh weight of the tissue sample (0.2 g).These concentrations were expressed as μg g^-1^ FW.

(1) $$\text{Chlorophyll a }(\frac{\text{μg}}{\text{ml}})=12.25*\text{A}663.2-2.79*\text{A}646.8\;$$

(2) $$\text{Chlorophyll b }(\frac{\text{μg}}{\text{ml}})=\;21.50*\text{A}646.8\;-\;5.1*\text{A}663.2$$

(3) $$\text{Carotenoid }(\frac{\text{μg}}{\text{ml}})=\frac{\left(1000+\text{A}470\;-1.8*\text{chla}-85.02*\text{chlb}\right)}{198}$$

#### 2.7.2 Total flavonoids

Total flavonoid content was measured following the method of (Chang et al., 2002). A 0.2 g ground sample was homogenized with 2.5 mL of 95% *(v/v)* methanol. The mixture was centrifuged at 13,000 g for 10 min, and 500 μL of the supernatant was transferred to a new tube. To this, 1.5 mL of 95% *(v/v)* methanol, 0.1 mL of 10% aluminum chloride (AlCl^3^), 0.1 mL of 1 M potassium acetate, and 2.8 mL of distilled water were added. The mixture was vortexed and incubated at room temperature for 30 min before measuring absorbance at 415 nm against a blank. Flavonoid content was estimated using a quercetin standard curve and expressed as mg g^-1^FW.

#### 2.7.3 Total phenolics

Total phenolic content (TPC) was determined using the Folin–Ciocalteu assay as described by (Ainsworth and Gillespie, 2007). A 0.2 g ground sample from each treatment was homogenized in 2 mL of ice-cold 95% *(v/v)* methanol and incubated in the dark at room temperature for 48 h. The mixture was then centrifuged at 13,000 g for 5 min, and 100 μL of the supernatant was transferred to a new microfuge tube. Next, 200 μL of 10% Folin–Ciocalteu reagent was added and vortexed for 5 min. Following this, 800 μL of 700 mM sodium carbonate was added, vortexed for 1 min, and incubated at 25 °C for 2 h. The absorbance of the resulting mixture was measured at 765 nm. Total phenolic content was calculated using a gallic acid equivalents standard curve and expressed as mg gallic acid equivalents per gram of sample.

### 2.8 Statistical analysis

The effects of AsA seed-priming treatments (four levels) on yield, biochemical, and nutritional responses were analyzed using one-way analysis of variance (ANOVA). Mean separation was performed using Fisher’s least significant difference (LSD) test at the 5% significance level for response variables with significant differences. Assumptions of normality, homogeneity of variance, and independence were verified prior to analysis, and no data transformation was required for these variables. All analyses were conducted using Minitab (version 19.0, Minitab Inc., State College, PA, USA).

Morpho-physiological variables measured repeatedly from Weeks 1 to 7 after transplanting were analyzed using a repeated measures mixed-model approach in SAS (SAS Institute Inc., 2016). The model evaluated the fixed effects of AsA treatment, sampling week, and their interaction. Models were fitted using restricted maximum likelihood (REML), which allows the analysis of unbalanced repeated-measures data using all available observations without requiring imputation or listwise deletion. In 2025, gas exchange data for the first four sampling weeks were unavailable because of a temporary equipment failure. As this failure was unrelated to treatment or plant condition and affected all treatments equally, the missing observations were considered consistent with the missing at random assumption and were retained as missing within the mixed model analysis rather than being imputed. Candidate covariance structures for the repeated-measures analysis, including compound symmetry, first-order autoregressive, and unstructured structures, were compared using the Akaike Information Criterion (AIC) (Littell et al., 1998). AIC values for all candidate covariance structures are presented in **Supplementary Table 2**. The PROC MIXED procedure was used to analyze fixed effects, and least squares mean (LSMEANS) with Fisher’s LSD adjustment were used for multiple comparisons and letter grouping. Model residuals were extracted and evaluated to verify that the assumptions underlying the analysis were satisfied. Residual normality was assessed using the Shapiro–Wilk test, while homogeneity of variance was evaluated by examining residual-versus-predicted value plots (Montgomery, 2017). For variables that did not initially satisfy these assumptions, appropriate transformations were applied prior to analysis: a cube root transformation for plant height, square root transformation for stem girth, and square transformation for leaf area. These transformations improved normality and stabilized variance, ensuring the validity of statistical inference. For clarity, figures present back-transformed means, while all statistical analyses and inferences were conducted on the transformed scale. Results are presented as back-transformed means ± standard error (SE), and statistical significance was declared at p < 0.05.

## 3 Results

### 3.1 Morpho-physiological response

AsA seed priming and sampling weeks significantly (p < 0.05) influenced plant growth and physiological parameters (**Table 1**; **Supplementary Table S3**). Treatment effects were significant (p < 0.001), across all measured traits, with particularly strong responses observed for net photosynthesis rate (Pn), stomatal conductance (Gs), and chlorophyll index (CI) while leaf area (LA) and transpiration (E) showed significance at p < 0.01. Weeks had a highly significant effect (p < 0.001) on all parameters, indicating strong temporal variation in plant responses. The interaction between treatment and weeks was mostly non-significant. A significant treatment × week interaction was observed for Pn (p < 0.05), indicating that the magnitude of the AsA treatment effect on net photosynthesis differed among sampling weeks. However, the relative ranking of treatments remained consistent across weeks (**Figure 3**).

**Table 1:** Significance levels (ANOVA) for the effects of ascorbic acid priming, time, and their interaction on morpho-physiological parameters of broccoli.

Effect	H	CL	G	LA	Pn	Gs	E	CI
Treatment (T)	*	*	*	**	***	***	**	***
Weeks (W)	***	***	***	***	***	***	***	***
T*W	ns	ns	ns	ns	*	ns	ns	ns

Plant height (H), canopy length (CL), Stem girth (G), leaf area (LA), net photosynthesis rate (Pn), stomatal conductance (Gs), transpiration (E) and chlorophyll index (CI). Significance levels are indicated as p < 0.05 (*), p < 0.01 (**), and p < 0.001 (***); non-significant differences are denoted as ns.

Net photosynthesis of broccoli plants was significantly (p < 0.05) influenced by seed-priming treatment and sampling week (**Table 1**). The heatmap illustrates a clear effect of AsA seed priming on the Pn across weeks and concentrations (**Figure 3**). Pn was consistently lowest in the control and 0 mgL^-1^ treatments across all weeks, ranging from 2.7 to 7.4 µmol m^−2^s^−1^. In contrast, AsA priming at 1 mgL^-1^ and 10 mgL^-1^ maintained substantially higher Pn values throughout the experimental period, ranging from 6.5 to 13.6 µmol m^−2^s^−1^. The 10 mgL^-1^ treatment showed the greatest enhancement, with a peak value of 13.6 µmol m^−2^s^−1^ at week 5, compared with 4.5 µmol m^−2^s^−1^ in the control, representing an increase of approximately 67%. Across the experimental duration, Pn increased from early weeks to a peak around weeks 4–5, followed by a slight decline towards week 7. Overall, AsA priming at 1 and 10 mgL^-1^ consistently enhanced photosynthetic performance, with the strongest effects observed at 10 mgL^-1^ across the experimental period.

AsA seed priming significantly (p < 0.05) influenced plant growth parameters compared to both control and water (0 mgL^-1^) treatments (**Figure 4**). Plant height significantly (p < 0.05) increased with AsA concentration. The 10 mgL^-1^ primed plants were tallest, representing a 17.4% increase over the control and a 12.5% increase over 0 mgL^-1^. Similarly, canopy length was significantly (p < 0.05) increased at 10 mgL^-1^, corresponding to a 19.7% increase over the control and a 15.4% increase over 0 mgL^-1^. Stem girth followed a similar trend, with 10 mgL^-1^ showing a 51.7% increase over the control and a 33.3% increase over 0 mgL^-1^. Leaf area was significantly (p < 0.01) influenced by AsA priming, with 10 mgL^-1^ resulting in a 69.4% increase over the control and a 51.7% increase over 0 mgL^-1^. It is crucial to note that the 1 mgL^-1^ treatment also demonstrated a significant (p < 0.01) improvement, with a 46.2% increase over the control. This increased growth parameters trend was visually evident as shown in **Figure 5**. All the measured growth parameters were significantly (p < 0.001) influenced by weeks, indicating strong temporal effects on plant development. A consistent increasing trend was observed across weeks, with leaf area showing the greatest variability and a peak around week 6, followed by a slight decline, while other parameters continued to increase through week 7 (**Figure 6**). Overall, AsA treatments consistently enhanced growth traits compared to both control and water treatments, with the strongest responses observed at 10 mgL^-1^.

**Figure 4:**
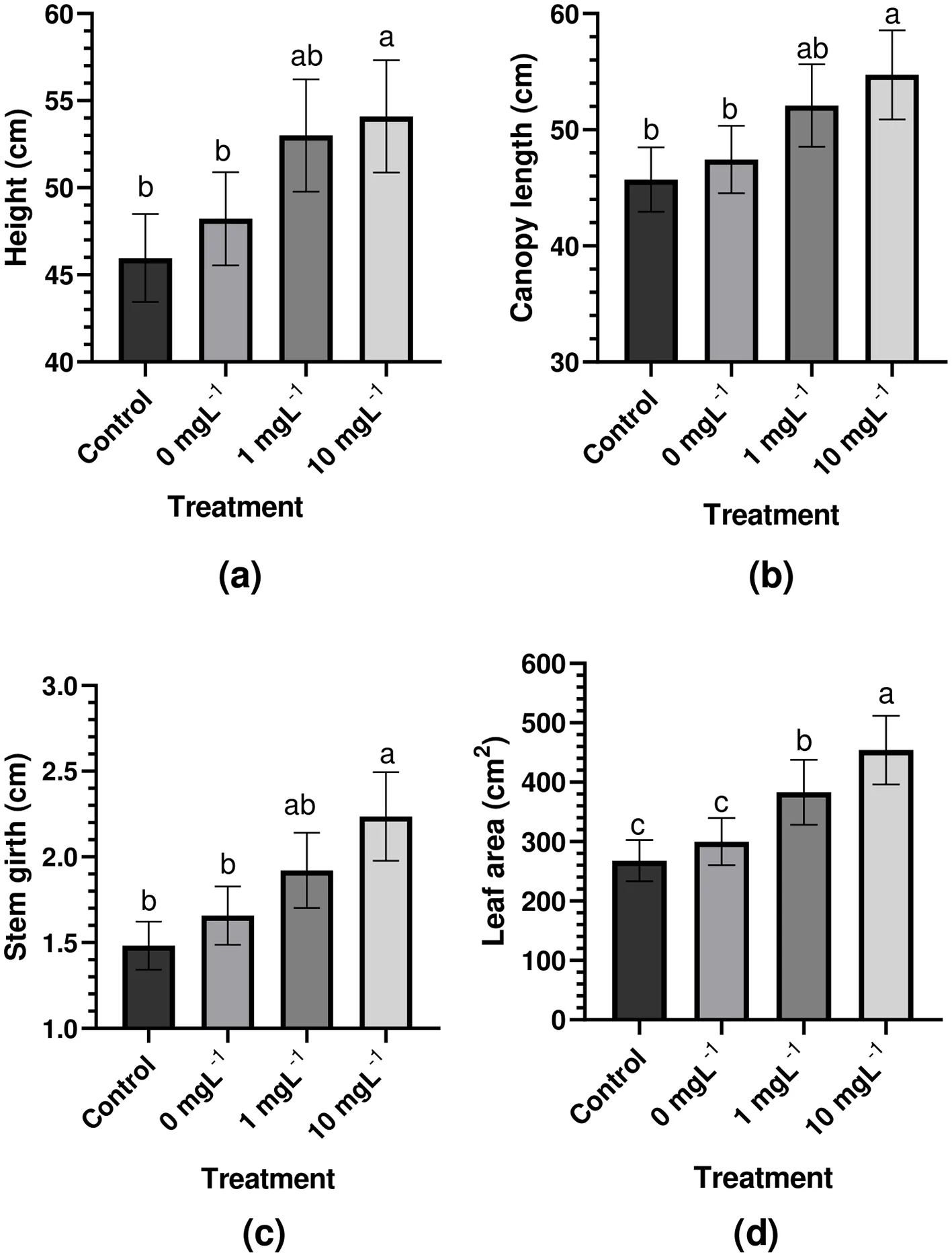
Main effects of AsA-treated broccoli on **(a)** plant height, **(b)** canopy length, **(c)** stem girth, and **(d)** leaf area. Each bar represents the mean calculated from 28 data points (4 site-year blocks × 7 sampling weeks), and error bars indicate the standard error. Means with different letters are significantly different at p < 0.05, as determined by Fisher’s least significant difference (LSD) test.

**Figure 5:**
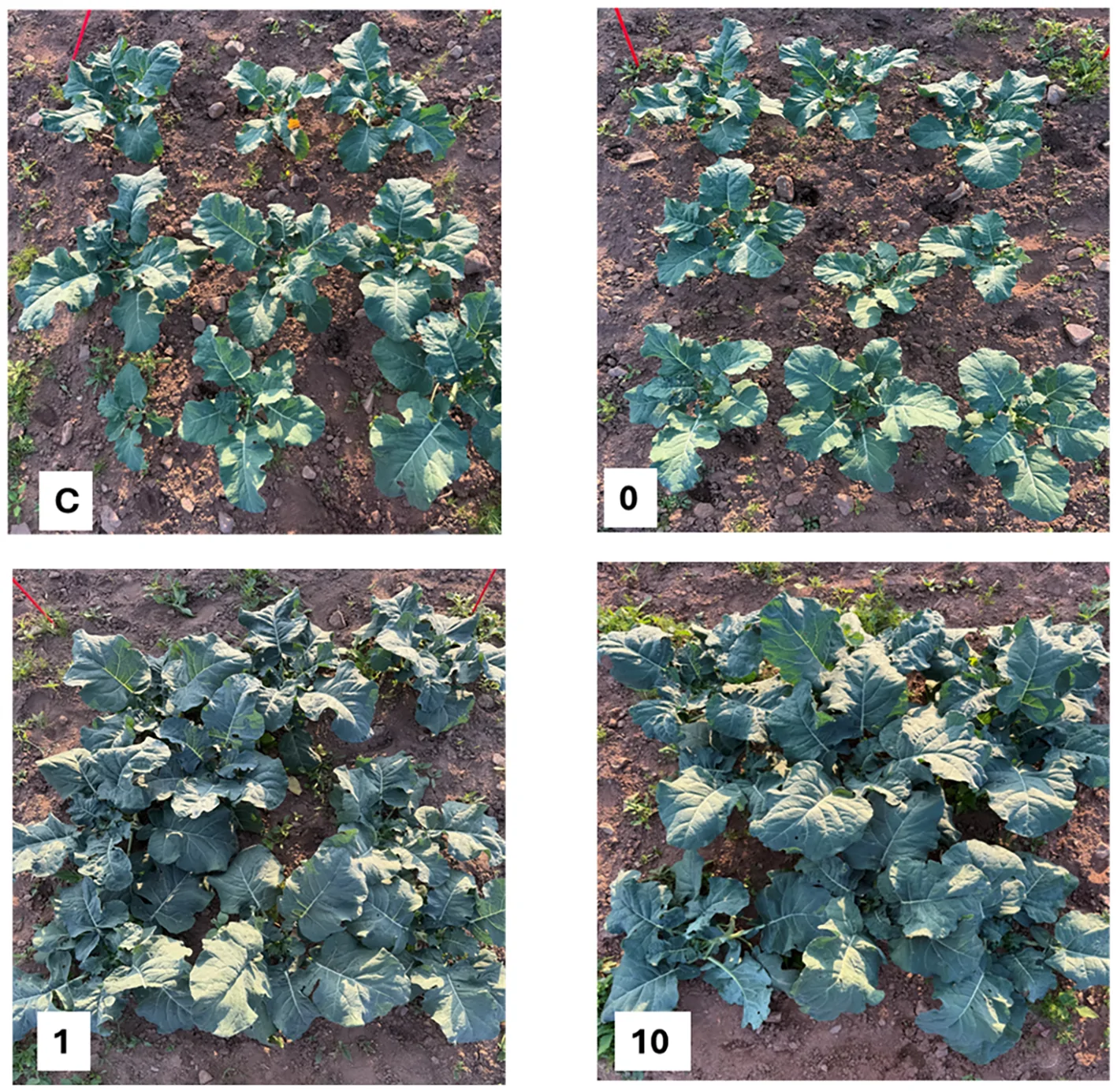
Overhead view of broccoli canopy four weeks after transplanting in the Chef’s Garden, Truro, Nova Scotia. Control seedlings (C) received no seed priming, while the treated groups were preconditioned with 0 mg L^-1^, 1 mg L^-1^, or 10 mg L^-1^ AsA.

**Figure 6:**
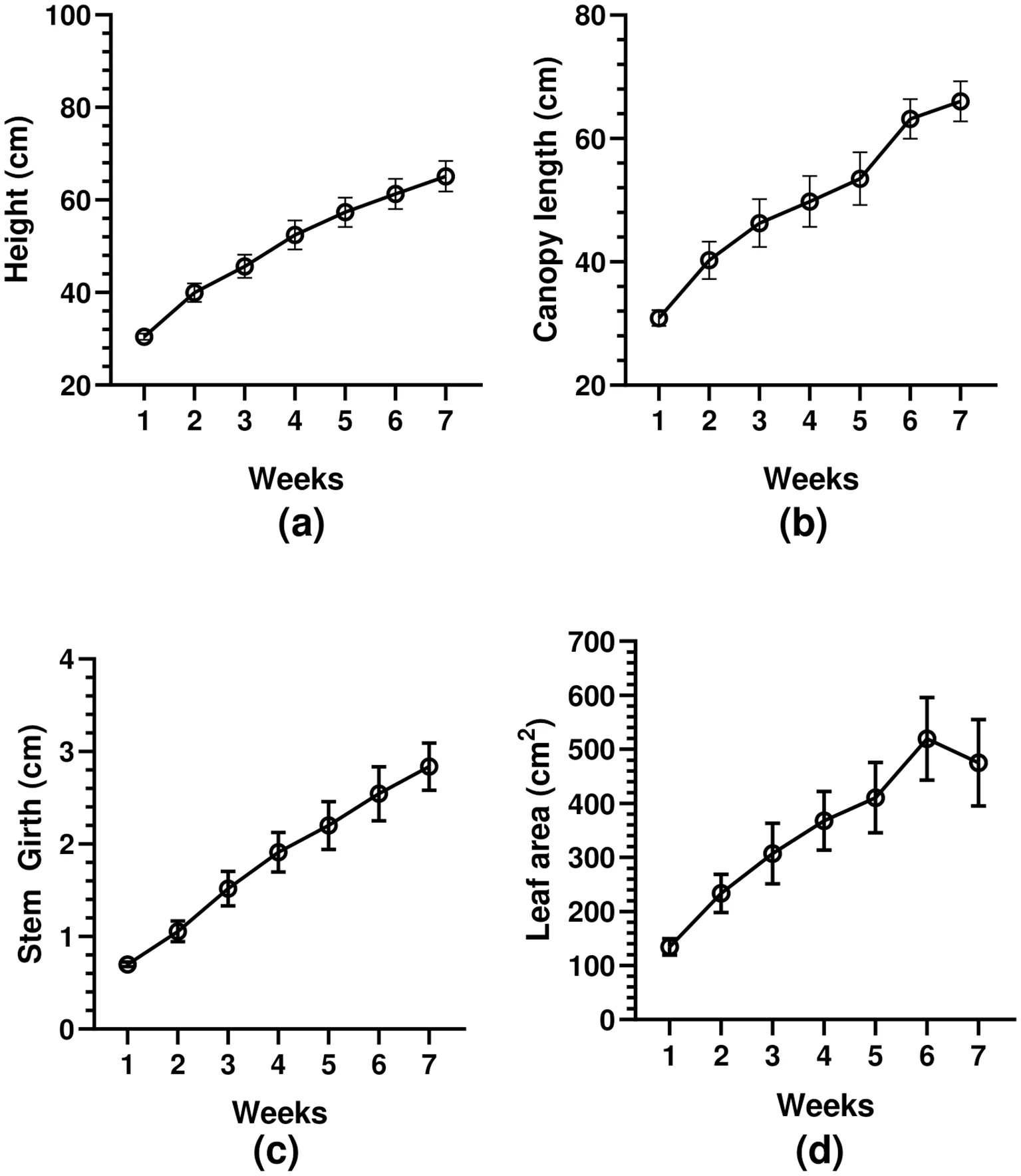
Main effects of sampling weeks on **(a)** plant height, **(b)** canopy length, **(c)** stem girth, and **(d)** leaf area. Each data point represents the mean calculated from 16 data points (4 AsA treatments × 4 site-year blocks), and error bars indicate the standard error. Statistical significance was evaluated using Fisher’s least significant difference (LSD) test at p < 0.05.

AsA seed priming significantly (p < 0.001) influenced Gs and E. Gs significantly (p < 0.001) increased with AsA concentration, with 10 mgL^-1^ showing the highest value, representing a 48.1% increase over control and a 35.6% increase over 0 mg/L. The 1 mgL^-1^ treatment also showed improvement, corresponding to a 18.5% increase over control, although it was not significantly different from 0 mgL^-1^. Similarly, E significantly (p < 0.001) increased with AsA treatment, with 10 mg/L showing a 31.8% increase over control and a 26.1% increase over 0 mgL^-1^. The 1 mgL^-1^ treatment was not significantly different from the control and 0 mg/L treatments (**Figure 7**). Chlorophyll index (CI) was significantly (p < 0.001) influenced by AsA seed priming (**Figure 8**). The highest CI was observed at 10 mgL^-1^, followed by 1 mgL^-1^, both of which were significantly (p < 0.001) higher than the control and 0 mgL^-1^ treatments. This represents an approximate 22.2% increase over control and a 15.8% increase over 0 mg/L at 10 mgL^-1^ (**Figure 8**). Gs, E and CI were significantly (p < 0.001) influenced by sampling weeks, indicating strong temporal variation in physiological responses. Gs exhibited a fluctuating trend, increasing up to week 2, declining in week 3, rising again during weeks 4 and 5, followed by a decline in week 6 and a slight increase in week 7. In contrast, E showed peak values in weeks 1 and 4, followed by a gradual decline in the subsequent weeks (**Figure 9**). CI showed an increasing trend from week 1 to week 7, with a noticeable increase to week 5, a slight decline in week 6, and a subsequent increase reaching the highest value in week 7 (**Figure 10**). Overall, AsA priming improved plant physiological performance, with the greatest and most consistent effects observed at 10 mgL^-1^.

**Figure 7:**
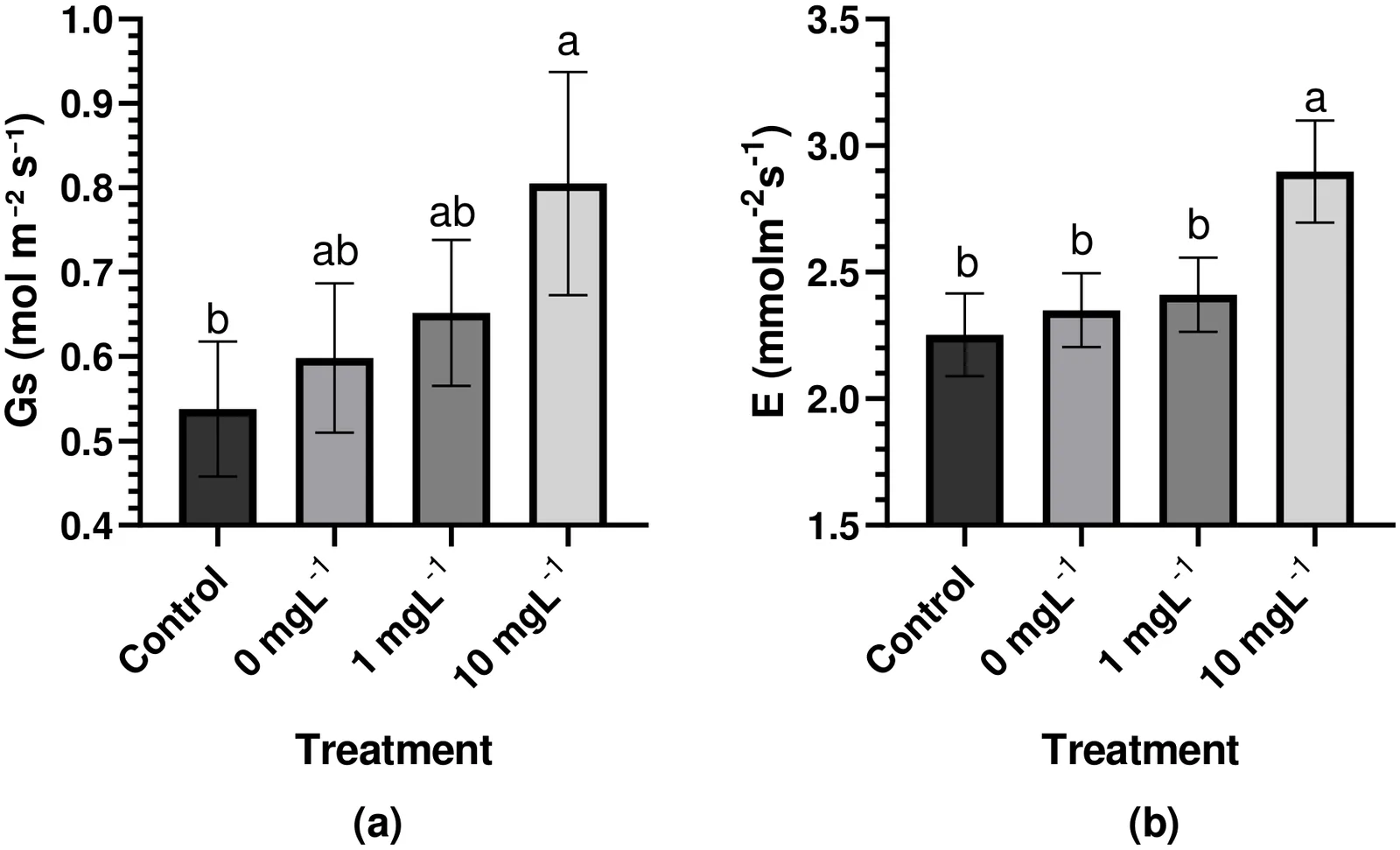
Main effects of AsA-treated broccoli on **(a)** Stomatal conductance, and **(b)** Transpiration. Each bar represents the mean calculated from Each bar represents the mean calculated from 28 data points (4 site-year blocks × 7 sampling weeks), and error bars indicate the standard error. Means with different letters are significantly different at p < 0.05, as determined by Fisher’s least significant difference (LSD) test.

**Figure 8:**
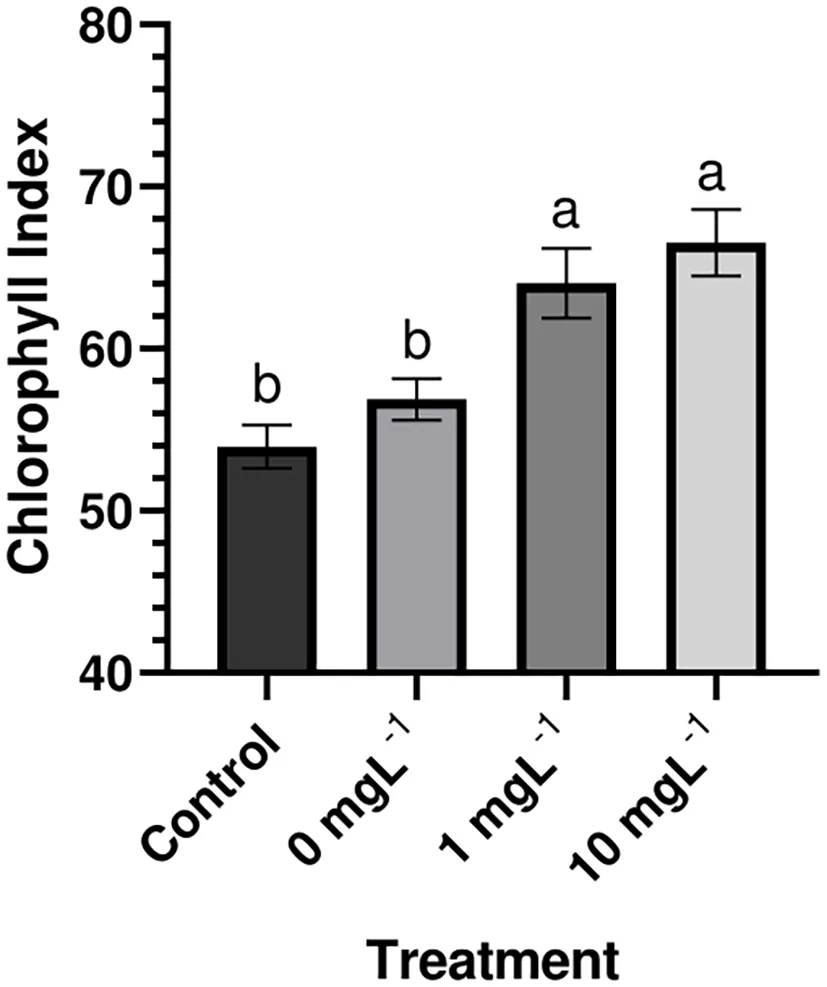
Main effects of AsA-treated broccoli on chlorophyll index. Each bar represents the mean calculated from 28 data points (4 site-year blocks × 7 sampling weeks), and error bars indicate the standard error. Means with different letters are significantly different at p < 0.05, as determined by Fisher’s least significant difference (LSD) test.

**Figure 9:**
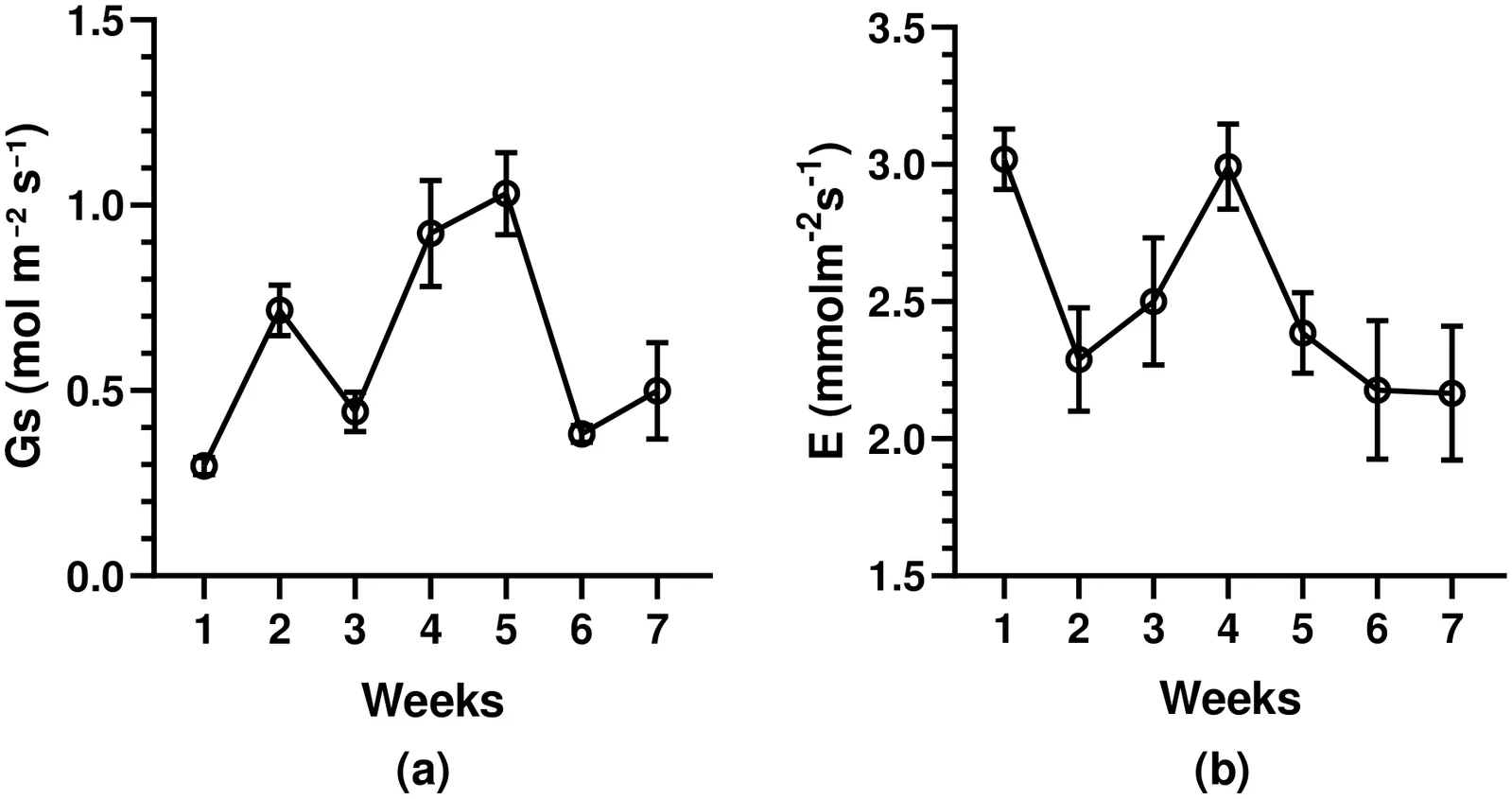
Main effects of sampling weeks on **(a)** Stomatal conductance, and **(b)** Transpiration. Each data point represents the mean calculated from 16 data points (4 AsA treatments × 4 site-year blocks), and error bars indicate the standard error. Statistical significance was evaluated using Fisher’s least significant difference (LSD) test at p < 0.05.

**Figure 10:**
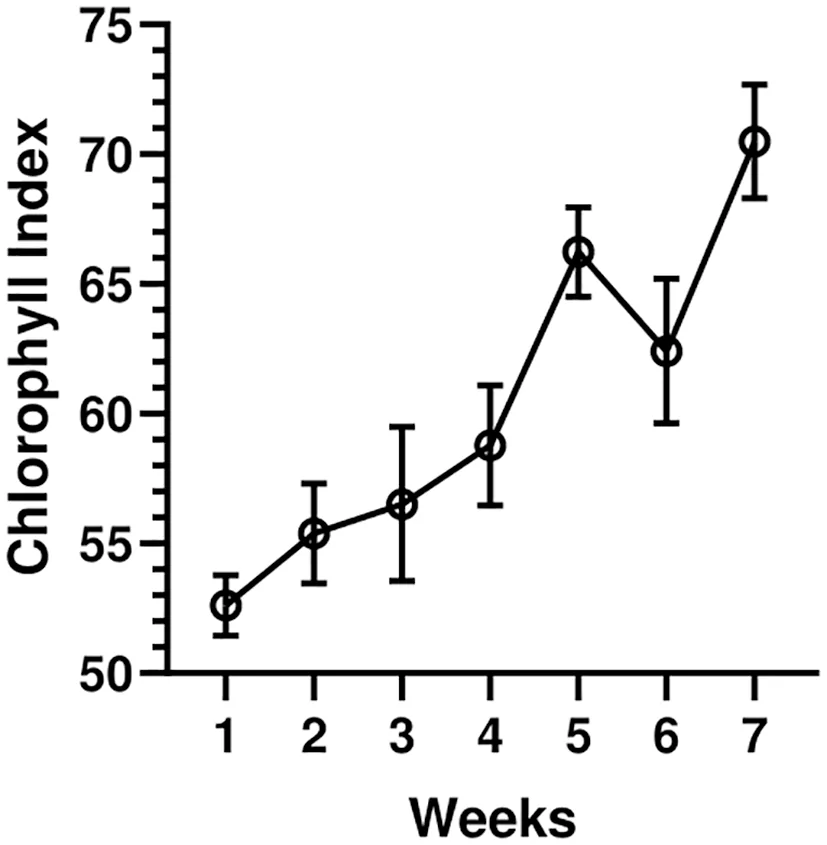
Main effects of sampling weeks on Chlorophyll index. Each data point represents the mean calculated from 16 data points (4 AsA treatments × 4 site-year blocks), and error bars indicate the standard error. Statistical significance was evaluated using Fisher’s least significant difference (LSD) test at p < 0.05.

### 3.2 Biochemical and nutritional response

AsA seed priming significantly (p < 0.05) influenced chlorophyll and carotenoid content, while no significant effects were observed for chlorophyll b, phenolics, and total flavonoids (**Table 2**). Chlorophyll a significantly (p < 0.05) increased with AsA concentration, with 10 mgL^-1^ showing the highest value, representing a 21.3% increase over the control and a 28.8% increase over 0 mgL^-1^. The 1 mgL^-1^ treatment also showed a significant improvement, corresponding to a 25.6% increase over 0 mgL^-1^, but it was not significantly different when compared to the control. Carotenoid content was also significantly (p < 0.05) enhanced by AsA priming, with 10 mgL^-1^ resulting in a 20.8% increase over the control and a 22.3% increase over 0 mgL^-1^. The 1 mgL^-1^ treatment followed a similar trend and showed a significant (p < 0.05) increase of 21.6% compared to 0 mgL^-1^ and was not significantly different from 10 mgL^-1^ and the control. In contrast, chlorophyll b, phenolics, and total flavonoids were not significantly affected by AsA treatments, although slight numerical increases were observed at 1 and 10 mgL^-1^ compared to the control and 0 mgL^-1^. Overall, AsA priming improved key photosynthetic pigments, particularly chlorophyll a and carotenoids, with the strongest effects observed at higher concentrations.

**Table 2:** Changes in chlorophylls, carotenoids, phenolics, and flavonoids in broccoli heads in response to AsA seed-priming treatments.

AsA treatment	Chlorophyll a (µg g^-1^)	Chlorophyll b (µg g^-1^)	Carotenoids (µg g^-1^)	Phenolics (mg QE g^-1^)	Total flavonoids (mg g^-1^FW)
Control	3.10 ± 0.20bc	2.27 ± 0.14	1.68 ± 0.15bc	526.20 ± 21.16	606.21 ± 36.01
0 mgL^-1^	2.92 ± 0.14c	2.04 ± 0.11	1.66 ± 0.20c	516.20 ± 23.18	538.78 ± 33.06
1 mgL^-1^	3.67 ± 0.30ab	2.47 ± 0.16	2.02 ± 0.22ab	571.22 ± 23.30	630.27 ± 39.16
10 mgL^-1^	3.76 ± 0.36a	2.46 ± 0.19	2.03 ± 0.25a	578.08 ± 31.79	544.20 ± 35.12
Significance level	*	ns	*	ns	ns

Values are expressed as means ± SE of five replicates. Where the ANOVA identified a significant effect (P< 0.05), means within a column followed by the same letter are not significantly different. Significance levels are indicated as follows: p < 0.05 (*), non-significant differences are denoted as ns.

AsA seed priming had no significant effect on most macro- and micronutrient concentrations in broccoli heads, including nitrogen (N), calcium (Ca), potassium (K), magnesium (Mg), sodium (Na), boron (B), copper (Cu), iron (Fe), manganese (Mn), and zinc (Zn) (**Table 3**). Nutrient levels remained relatively stable across all treatments, indicating that AsA application did not markedly alter overall nutrient accumulation in plant tissues. However, phosphorus (P) content was significantly (p < 0.05) influenced by AsA treatment. The control exhibited the highest P concentration, which was significantly (p < 0.05) higher than the 10 mgL^-1^ and 1 mgL^-1^, representing a 3.9% and 1.3% decrease respectively. The 1 mgL^-1^ treatment showed an intermediate response and was not significantly different from the control, while 0 mgL^-1^ was significantly (p < 0.05) lower than the control and 1 mgL^-1^. Overall, AsA priming had no impact on broccoli head nutrient composition, with the exception of phosphorus, where slight but significant variations were observed among treatments.

**Table 3:** Changes in elemental composition of broccoli ‘Gypsy’ head in response to AsA treatments.

Element	AsA Treatment				
	Control	0 mgL^-1^	1 mgL^-1^	10 mgL^-1^	Significance level
Nitrogen (%)	5.92 ± 0.23	5.65 ± 0.18	5.62 ± 0.17	5.51 ± 0.20	ns
Calcium (%)	0.31 ± 0.02	0.31 ± 0.02	0.31 ± 0.02	0.31 ± 0.02	ns
Potassium (%)	3.22 ± 0.04	3.13 ± 0.03	3.20 ± 0.03	3.14 ± 0.03	ns
Magnesium (%)	0.21 ± 0.00	0.20 ± 0.00	0.21 ± 0.00	0.21 ± 0.00	ns
Phosphorus (%)	0.77 ± 0.02a	0.75 ± 0.02bc	0.76 ± 0.02ab	0.74 ± 0.02c	*
Sodium (%)	0.04 ± 0.00	0.04 ± 0.00	0.04 ± 0.00	0.04 ± 0.00	ns
Boron (mgL^−1^)	24.94 ± 1.41	24.76 ± 1.20	26.05 ± 1.17	24.76 ± 1.04	ns
Copper (mgL^−1^)	6.65 ± 0.15	6.45 ± 0.16	6.45 ± 0.18	6.74 ± 0.25	ns
Iron (mgL^−1^)	73.74 ± 3.10	69.59 ± 2.47	69.87 ± 2.30	68.55 ± 1.88	ns
Manganese (mgL^-1^)	26.66 ± 0.77	28.77 ± 1.95	27.25 ± 0.71	27.44 ± 0.74	ns
Zinc (mgL^−1^)	75.47 ± 10.25	61.56 ± 3.12	61.52 ± 3.35	61.43 ± 3.21	ns

Values are expressed as means ± SE of five replicates. Where the ANOVA identified a significant effect (P< 0.05), means within a row followed by the same letter are not significantly different. Significance levels are indicated as follows: p < 0.05 (*), non-significant differences are denoted as ns.

### 3.3 Yield response

AsA seed priming significantly (p <0.05) influenced primary yield, secondary yield, and the number of secondary heads per plant (**Figures 11**, **12**). Primary yield significantly (p<0.001) increased with AsA concentration, with 10 mgL^-1^ showing the highest value (457.8 ± 23.1 g), representing a 38.4% increase over control and a 34.4% increase over 0 mgL^-1^. The 1 mgL^-1^ treatment also showed an increase over control, but it was not significantly different from the control and 0 mgL^-1^ treatments. This trend is also visually evident in **Figure 13**, where broccoli heads from AsA-treated plants (1 and 10 mgL^-1^) appear larger compared to the control and water-primed treatments. Similarly, secondary yield was significantly (p < 0.05) affected by AsA treatment, with 10 mgL^-1^ exhibiting the highest value, representing an 80.4% increase over the control and a 72.0% increase over 0 mgL^-1^. The number of secondary heads per plant was also significantly (p < 0.05) influenced by AsA priming. The highest values were observed at 1 mgL^-1^ and 10 mgL^-1^, representing increases of 15.3% and 10.6% over the control, respectively, and 40.2% and 35.0% over 0 mgL^-1^. It is important to note that water priming (0 mgL^-1^) resulted in lower values than the control and other AsA treatments. Additionally, dry matter percentage was not significantly affected by AsA treatment. Overall, AsA priming improved yield components, including primary yield, secondary yield, and the number of secondary heads.

**Figure 11:**
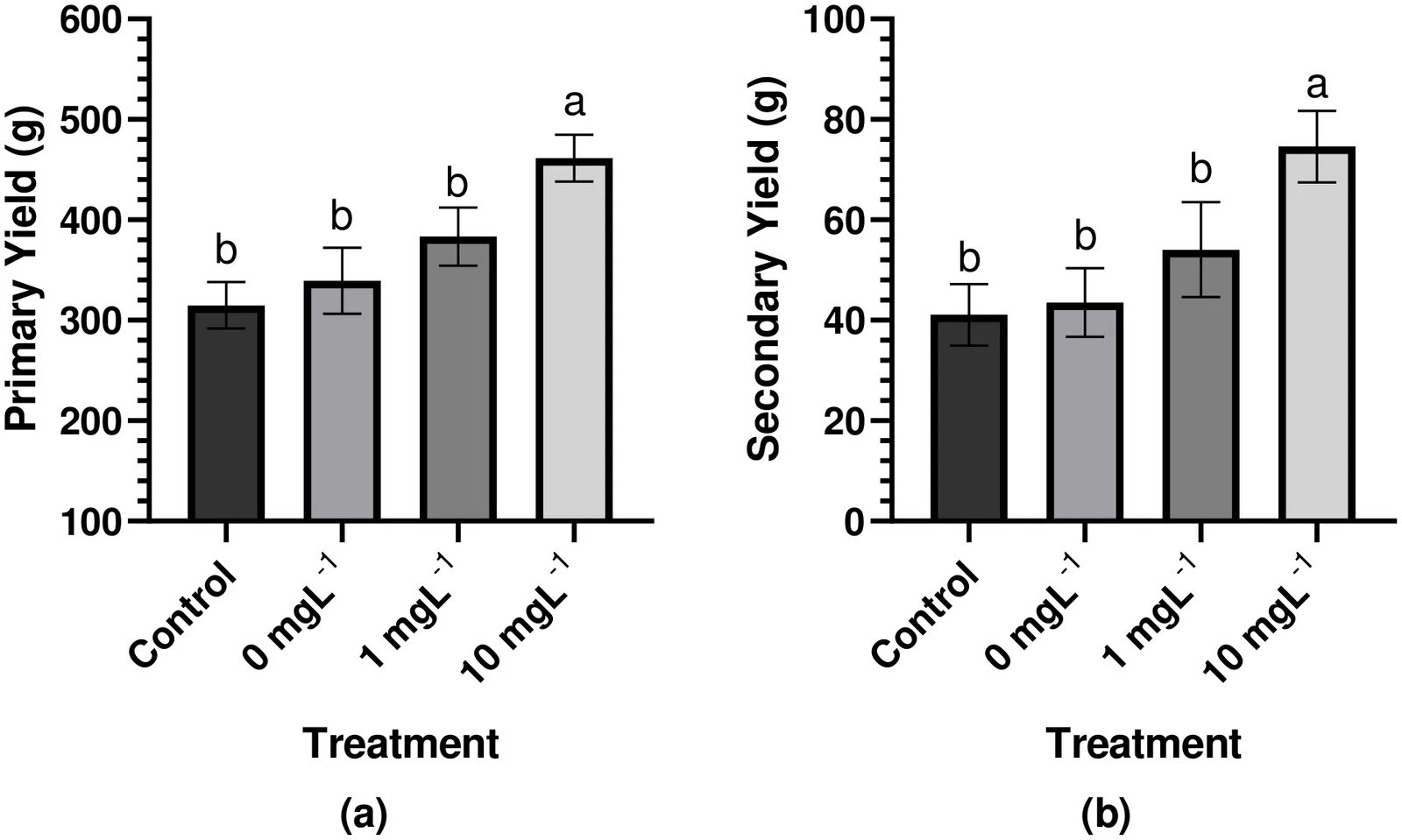
Effect of AsA treatment on **(a)** primary yield per plant (g) and **(b)** secondary yield per plant (g). Bars represent means, and error bars indicate the standard error. Means with different letters are significantly different at p < 0.05, as determined by Fisher’s least significant difference (LSD) test.

**Figure 12:**
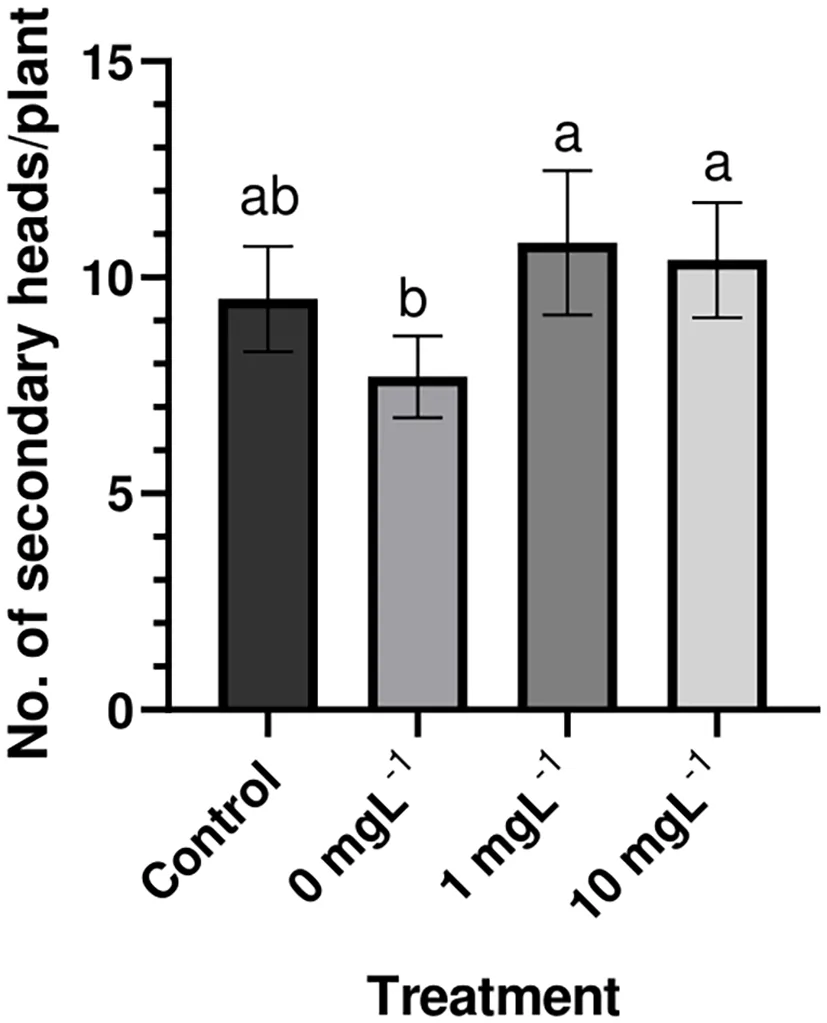
Effect of AsA treatment on number of secondary heads. Bars represent means, and error bars indicate the standard error. Means with different letters are significantly different at p < 0.05, as determined by Fisher’s least significant difference (LSD) test.

**Figure 13:**
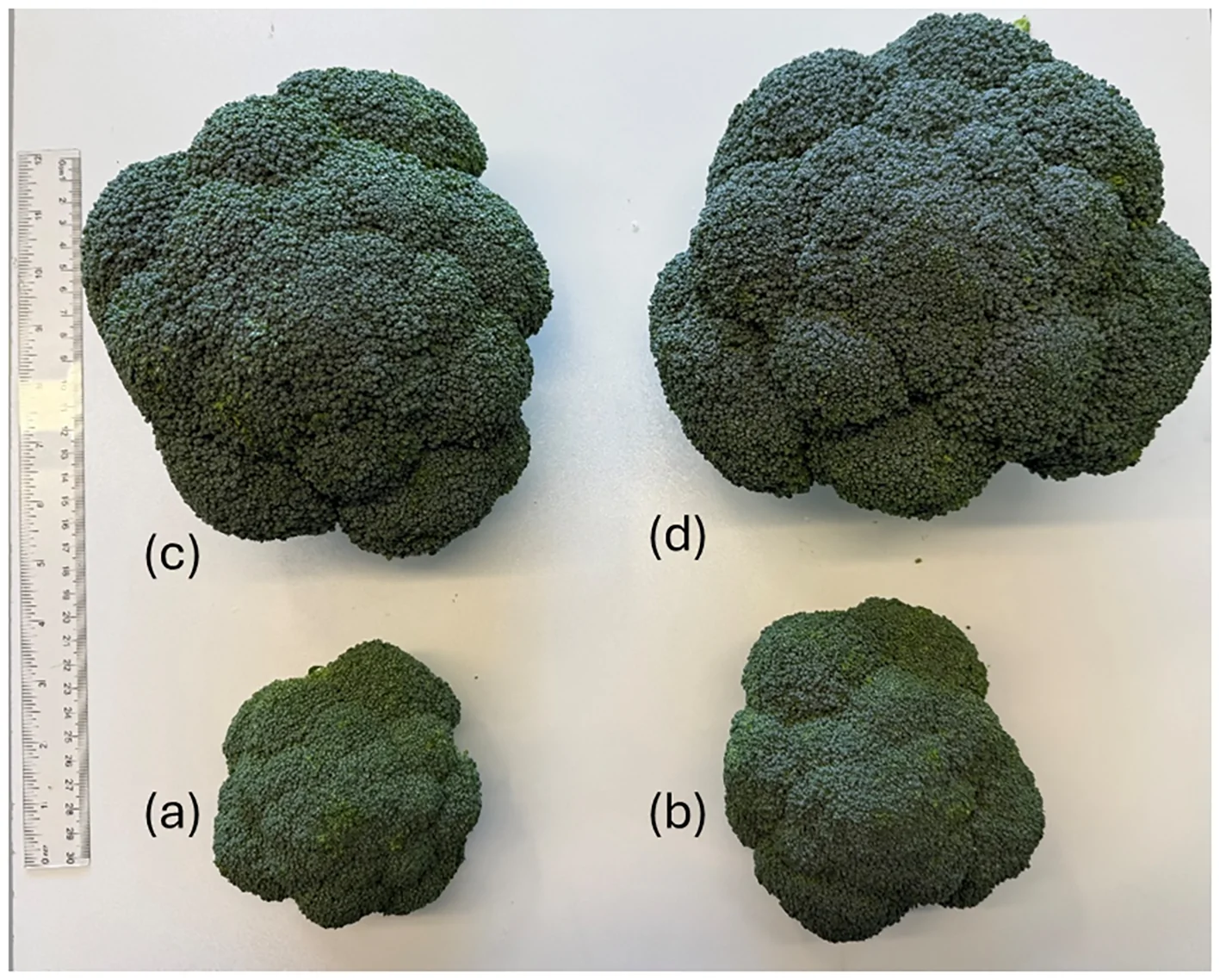
Representative primary broccoli heads from each treatment, harvested at the same maturity stage. **(a)** Control; **(b)** 0 mg L^−1^ AsA; **(c)** 1 mg L^−1^ AsA; **(d)** 10 mg L^−1^ AsA. Heads shown are representative of each treatment and do not necessarily correspond to treatment means.

## 4 Discussion

Seed priming using AsA substantially improved multiple growth, physiological, and yield attributes of broccoli cv. Gypsy under field conditions. The findings align with earlier reports demonstrating AsA as an efficacious seed priming agent that enhances photosynthetic performance, biomass accumulation, and stress tolerance in a variety of crops, including broccoli, *Solanum lycopersicum* (tomato), *Triticum aestivum* (wheat), and *Chenopodium quinoa* (quinoa) (MacDonald et al., 2022; Cai et al., 2024; MacDonald et al., 2009; Shah et al., 2019, 2025). The present study extends these findings from controlled environments to field conditions, providing robust evidence that AsA priming can improve overall crop performance without detrimental effects on nutritional quality (**Figures 2**–**13**; **Tables 2**, **3**). AsA priming significantly (p<0.05) improved plant growth parameters, including plant height, canopy length, stem girth, and leaf area, which indicates that AsA priming promotes early vigor and sustained vegetative development. Such findings are consistent with previous studies reporting enhanced biomass accumulation and leaf expansion in AsA-treated plants (Cai et al., 2024; MacDonald et al., 2022; Shah et al., 2025), which can be attributed to AsA’s role in promoting cell division, cell elongation, and hormonal regulation. AsA serves as a key redox buffer and acts as a cofactor for enzymes involved in photosynthesis, hormone biosynthesis, and the regeneration of other antioxidants (Gallie, 2013). Additionally, it plays an important role in regulating cell growth and mediating signal transduction pathways (Gallie, 2013). The significant (p<0.05) increase in leaf area (up to 69.4%) and canopy length (up to 19.7%) observed in this study likely enhanced light interception by increasing the photosynthetically active surface area, thereby improving carbon assimilation capacity and supporting higher photosynthetic activity (Retta et al., 2024; Duursma et al., 2012).

Gas exchange emerged as a key response variable in this study. Pn, Gs, and E were significantly (p<0.05) enhanced by AsA priming, particularly at 10 mgL^-1^, indicating improved gas exchange and stomatal regulation. These findings are consistent with previous studies in quinoa and wheat (S. Shah et al., 2025; Baig et al., 2021). The increase in Gs and E suggests improved stomatal functionality, which may facilitate greater CO_2_ uptake and nutrient transport (Takahashi et al., 2025). The enhanced CI observed in this study supports the argument that AsA promotes chlorophyll biosynthesis by stimulating the formation of chlorophyll precursors, thereby contributing to increased photosynthetic activity which is consistent with findings reported in tomato (Chen et al., 2024). Although oxidative stress markers (e.g., H_2_O_2_, malondialdehyde) were not directly measured in this study, the improved photosynthetic performance is consistent with the established role of AsA as a key antioxidant within chloroplasts, where it protects the photosynthetic machinery from oxidative damage and maintains efficient electron transport (Chen et al., 2023).

AsA priming significantly increased chlorophyll and carotenoid concentrations, reflecting enhanced synthesis or reduced oxidative degradation of these pigments. Both pigment groups play central roles in photoprotection and photosynthetic efficiency; therefore, their accumulation suggests that AsA enhances antioxidant defenses within the chloroplast (Pérez-Gálvez et al., 2020). Chlorophyll a and chlorophyll b are the principal pigments involved in photosynthesis (Nurachman et al., 2015). Chlorophyll a serves as the primary light-harvesting pigment, capturing energy predominantly from the blue and red regions of the light spectrum and initiating photochemical reactions (Wang and Grimm, 2021), whereas chlorophyll b functions as an accessory pigment, extending the range of light absorption and transferring energy to chlorophyll a, thereby improving overall photosynthetic efficiency (Challabathula et al., 2016). In this study, AsA priming significantly (p<0.05) increased chlorophyll a, which confirms previous findings on broccoli, wheat, quinoa, and mung bean (Cai et al., 2024; Shah et al., 2025; Farook et al., 2012; Prajapati et al., 2025). As chlorophyll a is essential for electron transport, its stability is critical for maintaining efficient photochemical processes (Challabathula et al., 2016). In contrast, chlorophyll b was not significantly affected by AsA treatment, suggesting that AsA may preferentially influence enzymes or precursors involved in chlorophyll a biosynthesis or provide greater protection against its degradation. However, under stress conditions, AsA may exert broader effects on pigment accumulation. For instance, studies in pea under salt stress have reported substantial increases in both chlorophyll a (41.1%) and chlorophyll b (56.1%), indicating that AsA can enhance both pigments depending on environmental conditions (Kanwal et al., 2024).

AsA priming significantly (p < 0.05) increased carotenoid content, indicating that its effect on pigment enhancement was consistent under field conditions. This suggests that AsA may directly stimulate or stabilize carotenoid biosynthesis, thereby reducing ROS generation and protecting the photosynthetic machinery (T. Sun et al., 2022), which is consistent with previous studies in *Medicago polymorpha*, mung bean, quinoa, and broccoli under various stress conditions (Cai et al., 2024; S. Shah et al., 2025; Prajapati et al., 2025; Gharred et al., 2023). In addition to their physiological roles in plants, carotenoids contribute significantly to human health. These dietary pigments possess strong antioxidant properties that help reduce oxidative stress, support immune function, and lower the risk of chronic diseases, including cardiovascular disorders, certain cancers, and age-related eye conditions such as macular degeneration and cataracts (Fiedor and Burda, 2014). Interestingly, total phenolics and flavonoids were not significantly affected by AsA priming, suggesting that the observed growth promotion was not accompanied by major changes in secondary metabolite biosynthesis. Similar stability in phenolic composition following AsA priming has been reported in greenhouse-grown broccoli (Cai et al., 2024).

The mechanism underlying carotenoid enhancement under non-stress conditions remains to be established and may differ from that operating under stress. Under stress, carotenoid accumulation is closely coupled to abscisic acid (ABA) signaling, as carotenoids are precursors of ABA biosynthesis, and ABA, in turn, regulates the expression of carotenoid metabolic genes (Q. Sun et al., 2024). Although this study was conducted under non-stress conditions, carotenoid accumulation may still be influenced by basal hormonal regulation, including ABA, which plays a role in stomatal function and metabolic signaling (Liu et al., 2014). Given the close link between carotenoids and ABA biosynthesis, and the ability of ABA to regulate the expression of genes involved in carotenoid metabolism (Lado et al., 2019), hormone profiling and expression analysis of carotenoid pathway genes would be necessary to clarify the mechanisms underlying these responses.

Interestingly, total phenolic and flavonoid concentrations were not significantly affected by AsA treatment in this study. This response differs from that observed under stress conditions. In greenhouse seedling trials using the same priming protocol, drought stress increased flavonoid, phenolic, and hydrogen peroxide accumulation in non-primed seedlings, whereas AsA primed seedlings exhibited little or no comparable increase (Cai et al., 2024). These findings suggest that AsA priming may alleviate the physiological perception of stress rather than directly stimulate phenylpropanoid metabolism (Cohen and Kennedy, 2010; MacDonald and Mohan, 2025). Accordingly, because the accumulation of phenolic and flavonoid compounds in the greenhouse study appeared to be associated with the stress response rather than a constitutive effect of AsA, the absence of imposed stress under the present field conditions may explain the lack of significant treatment effects on these metabolites. This interpretation is consistent with the growth–differentiation balance theory, which proposes that, under conditions where growth is not constrained by resource limitation or environmental stress, assimilated resources are preferentially allocated to primary growth processes, including biomass accumulation and yield, rather than to differentiation-related processes such as secondary metabolite biosynthesis (Glynn et al., 2007). The concurrent increase in carotenoid concentration observed in the present study does not necessarily conflict with this theory, as carotenoids are integral components of the photosynthetic apparatus and are closely associated with chloroplast development, light harvesting, and photoprotection. Their accumulation may therefore reflect enhanced photosynthetic capacity and plant development rather than the induction of a generalized stress-related secondary metabolic response. Collectively, these findings indicate that AsA priming under non-stress field conditions preferentially enhanced processes associated with growth and photosynthetic function without inducing broad changes in phenolic and flavonoid metabolism, thereby revealing a response that differs from that observed under stress-imposed greenhouse conditions.

Enhanced photosynthetic activity and biomass accumulation can sometimes lead to reduced nutrient concentrations in plant tissues due to a dilution effect, where rapid carbon assimilation outpaces nutrient uptake (Kaur et al., 2025; Gojon et al., 2023). This phenomenon has been widely reported under conditions such as CO_2_ fertilization, where increased growth can result in lower concentrations of key minerals unless nutrient acquisition is proportionally increased (Kaur et al., 2025; Gojon et al., 2023). In the present study, nutrient analyses revealed no significant differences in macro- and micronutrient concentrations, except for a slight reduction in P (3.9% at 10 mg L^−1^). This marginal decline may be attributed to a dilution effect associated with increased biomass production or minor changes in root uptake dynamics (Shen et al., 2011). Importantly, other essential elements (N, K, Ca, Fe, Zn) remained unaffected, indicating that AsA priming did not disrupt nutrient homeostasis.

AsA seed priming resulted in significant (p<0.05) yield improvements, with primary yield increasing by up to 38.4% and secondary yield by 80.4% at 10 mgL^-1^ compared to the control. These findings are consistent with previous studies reporting yield enhancements in crops such as rice, wheat, and sunflower following AsA treatment (Davodi et al., 2019; Zhang et al., 2025; El-Hawary et al., 2023). The observed yield gains can be attributed to improved early vigor and enhanced leaf area, which likely supported greater light interception and photosynthetic efficiency. Increased photosynthetic activity contributed to enhanced vegetative growth, ultimately translating into higher yield, as illustrated in **Figure 14**. The observed increase in the number of secondary heads may be due to the sustained photosynthetic productivity late into the growing season. AsA priming primarily enhanced biomass accumulation without altering tissue composition or nutritional quality. This suggests that yield improvements were achieved without compromising produce quality. One important observation was that water-primed seeds occasionally produced lower yields than the non-primed control, suggesting that priming without an active agent may impose stress or reduce seed vigor under certain conditions. This contrasts with reports of beneficial effects of hydropriming in other studies (MacDonald and Mohan, 2025) and highlights the potential protective role of AsA during the priming process.

**Figure 14:**
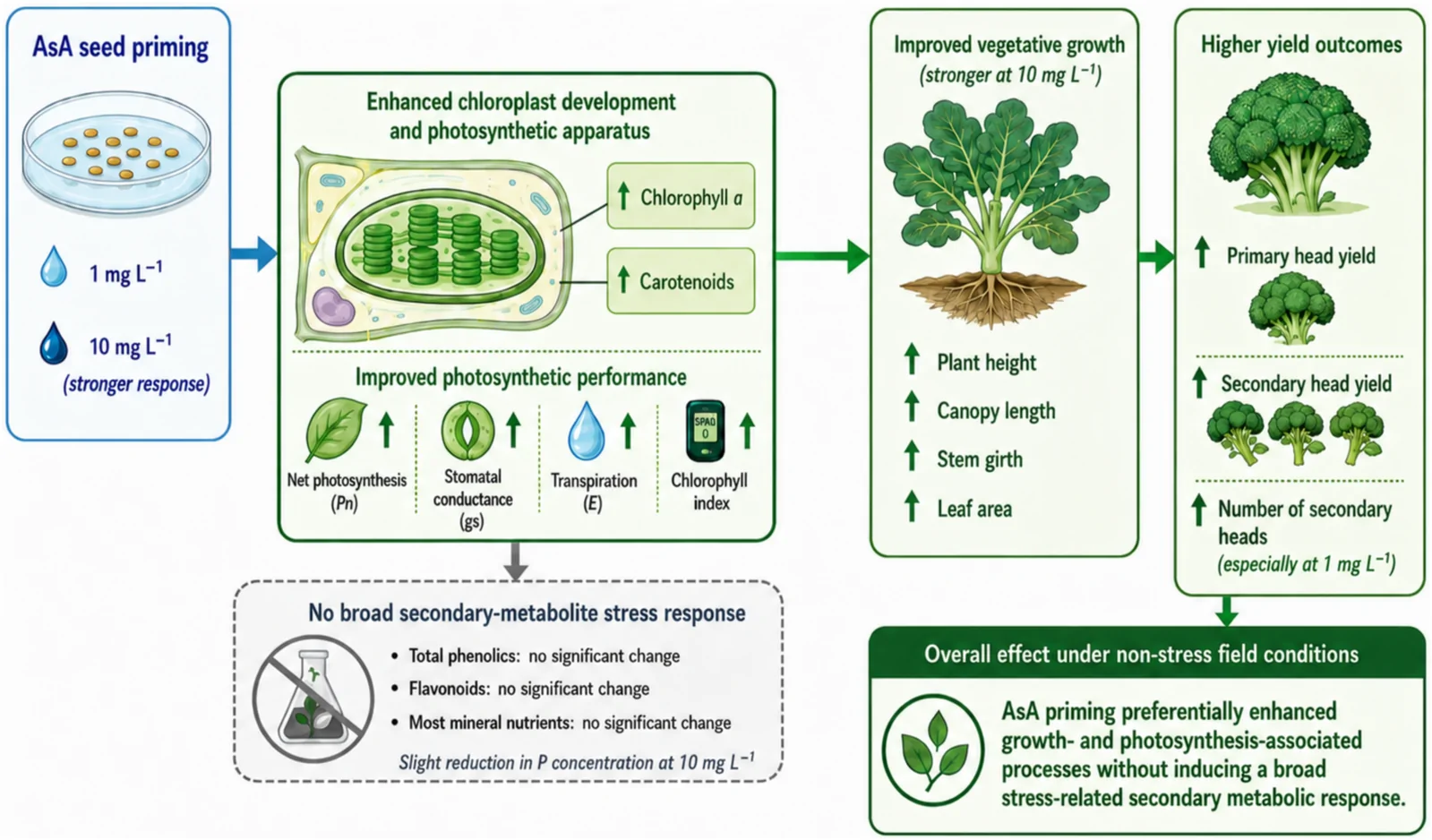
Proposed mechanism by which AsA seed priming enhances photosynthetic performance, vegetative growth, and yield of broccoli under non-stress field conditions.

A limitation of this study is that the pH of the priming solutions was not adjusted and decreased slightly with increasing AsA concentration, consistent with the inherent acidity of AsA in aqueous solution. Although this relatively narrow pH range was unlikely to independently affect seed performance (Vallabhaneni et al., 2025), the absence of a pH-matched, non-redox acid control means that a minor contribution of solution pH to the observed responses cannot be completely excluded. Future studies should incorporate such a control to more clearly distinguish pH-related effects from those specifically attributable to AsA. Future research should focus on integrative omics approaches, such as transcriptomic and metabolomic analyses, to identify key genes and metabolic pathways associated with AsA-mediated yield improvements and further elucidate the underlying mechanisms.

## 5 Conclusion

This two-year, multi-site field study demonstrates that AsA seed priming can enhance broccoli growth, physiological performance, and yield under field conditions. The findings extend previous controlled-environment research and support the practical application of AsA priming, particularly at 10 mg L^−1^, as a biologically effective and economically sustainable approach to improving crop productivity. Based on a reagent price of CAD 45 for 25 g of AsA, the cost was approximately CAD 1.80 g^−1^. At the 10 mg L^−1^ concentration used in this study, 100 mL of solution required to treat 50 seeds contained 1 mg of AsA, resulting in a very low direct reagent cost of approximately USD 0.0013 per 50 seeds. This indicates that the AsA input itself is inexpensive; however, labor, water, equipment, energy, handling, and commercial-scale processing costs were not assessed and should be considered when evaluating its economic feasibility at a larger scale. Under conditions similar to those evaluated in this study, priming broccoli seeds with 10 mg L^−1^ AsA for 24 h in darkness may be recommended to increase primary and secondary yield without additional fertilizer inputs or compromising nutritional quality.

## Data Availability

The original contributions presented in the study are included in the article/**Supplementary Material**. Further inquiries can be directed to the corresponding author.
