# Supplementary material for: Field evaluation of ascorbic acid seed priming on growth, physiology, yield, and nutritional quality of broccoli (Brassica oleracea L. var. italica ‘Gypsy’)
Source: Front Plant Sci. 2026 Sep 16;17:1897446. doi: 10.3389/fpls.2026.1897446 (PMC13624865; doi:10.3389/fpls.2026.1897446)
Supplement: Supplementary file 1 [file DataSheet3.pdf]

Soil Test Report

Department of Agriculture
Laboratory Services
PO Box 890
Harlow Institute
Truro, NS B2N 5G6

novascotia.ca/agriculture-labs
Tel: 902-893-6565
Fax: 902-893-4193

Client ID: C12258
Order ID: 2402032
Samples Reported: 26-Apr-24
Samples Received: 17-Apr-24
# of Samples Received: 2

DALHOUSIE UNIVERSITY - TRURO
FACULTY OF AGRICULTURE
PO BOX 550
TRURO, NS
B2N 5E3

Lab #	2402032-001			2402032-002								
Sample ID	1 - DEMO			2 - CHEF								
Crop to be Grown	Broccoli			Broccoli								
Parameter	Analysis		Rating	Analysis		Rating	Analysis		Rating	Analysis		Rating
Nitrogen (%)	0.15			0.17								
**pH (pH Units)**	6.32			6.73								
**Buffer pH (pH Units)**	7.79			7.82								
Organic Matter (%)	3.0			3.4								
**P2O5 (kg/ha)**	966		H+	1222		E						
**K2O (kg/ha)**	527		H	533		H						
**Calcium (kg/ha)**	2480		M	3696		M+						
**Magnesium (kg/ha)**	251		M	277		M						
**Sodium (kg/ha)**	13			15								
**Sulfur (kg/ha)**	23			25								
**Aluminum (ppm)**	1485			1347								
**Boron (ppm)**	< 0.50			< 0.50								
**Copper (ppm)**	1.57			1.00								
**Iron (ppm)**	126			138								
**Manganese (ppm)**	43			51								
**Zinc (ppm)**	3.44			4.08								
CEC (meq/100 g)	9.5			12.4								
Base sat. K (%)	5.9			4.5								
Base sat. Ca (%)	65.2			74.3								
Base sat. Mg (%)	11.0			9.3								
Base sat. Na (%)	0.3			0.3								
Base sat. H (%)	17.7			11.6								
**LR CaCO3 (t/ha to pH 6.5)**	1											
Required Nutrient	N	P2O5	K20	N	P2O5	K20	N	P2O5	K20	N	P2O5	K20
(kg/ha)	220	50	50	220	0	50						

Copies To:	Analysis Approved By:
	L. Scott.<br>Lori Scott, TLOS

Confidentiality Notice-This report is for the individual or Organization to whom it is addressed and contains confidential information. If received in error, please contact us immediately

Soil Test Report - Additional Information

- This report shall not be reproduced, except in full, without written approval of Laboratory Services
- **Bold** indicates analysis accredited to ISO/IEC 17025:2017 standard
- **[S]** Sample sent to an accredited lab for analysis
- **LA** indicates Lab Accident and the analysis was not able to be completed
- **LR** Lime Requirement
- The dates of performance of laboratory activity are available by contacting Laboratory Services at (902) 893-6565
- Result(s) apply to the sample as received, and relate only to the sample(s) tested

Conversions:

- 1 kg/ha = 0.89 lb/ac
- 1 tonne/ha = 0.45 ton/ac = 10 kg/100m^2
- 1 kg/ha/100 = 1 kg/100m^2
- to convert kg/ha to ppm divide by 2

Ratings:

- L = Low M = Medium H = High E = Excessive

Method References for Accredited Methods

- LSAL408 Determination of Mehlich III Extractable Major and Trace Metal Ions in Soil by ICP-OES (Modified Mehlich III, Commun. Soil. Sci. Plant Anal. 15(12): 1409- 1416)
- LSAL409 Determination of Soil Water pH and Lime Requirement using Adams-Evans Buffer by AS3010D pH Analyzer

The following Soil Related Factsheets are availabe on our website:
https://novascotia.ca/agriculture-labs

- How to take Field Soil Samples
- Understanding the Soils Test Report
