# Supplementary material for: Field evaluation of ascorbic acid seed priming on growth, physiology, yield, and nutritional quality of broccoli (Brassica oleracea L. var. italica ‘Gypsy’)
Source: Front Plant Sci. 2026 Sep 16;17:1897446. doi: 10.3389/fpls.2026.1897446 (PMC13624865; doi:10.3389/fpls.2026.1897446)
Supplement: Supplementary file 2 [file DataSheet4.pdf]

NOVA SCOTIA

Soil Test Report

Department of Agriculture
Laboratory Services
PO Box 890
Harlow Institute
Truro, NS B2N 5G6

novascotia.ca/agriculture-labs
Tel: 902-893-6565
Fax: 902-893-4193

Client ID: C12258
Order ID: 2503659
Samples Reported: 19-Jun-25
Samples Received: 17-Jun-25
# of Samples Received: 2

DALHOUSIE UNIVERSITY - TRURO
FACULTY OF AGRICULTURE
PO BOX 550
TRURO, NS
B2N 5E3

Lab #	2503659-001		2503659-002					
Sample ID	DEMO		CHEF					
Crop to be Grown	Broccoli		Broccoli					
Parameter	Analysis	Rating	Analysis	Rating	Analysis	Rating	Analysis	Rating
**pH (pH Units)**	6.00		6.56					
**Buffer pH (pH Units)**	7.72		7.79					
Organic Matter (%)	3.1		3.6					
**P2O5 (kg/ha)**	856	H	1145	E				
**K2O (kg/ha)**	400	H-	565	H+				
**Calcium (kg/ha)**	2252	M-	3724	M+				
**Magnesium (kg/ha)**	230	M	307	M+				
**Sodium (kg/ha)**	14		23					
**Sulfur (kg/ha)**	27		31					
**Aluminum (ppm)**	1419		1276					
**Boron (ppm)**	< 0.50		< 0.50					
**Copper (ppm)**	1.41		0.95					
**Iron (ppm)**	97		128					
**Manganese (ppm)**	23		27					
**Zinc (ppm)**	3.18		4.09					
CEC (meq/100 g)	9.3		12.9					
Base sat. K (%)	4.6		4.6					
Base sat. Ca (%)	60.7		72.1					
Base sat. Mg (%)	10.3		9.9					
Base sat. Na (%)	0.3		0.4					
Base sat. H (%)	24.1		13.0					
**LR CaCO3 (t/ha to pH 6.5)**	2							

Required Nutrient (kg/ha)	N	P2O5	K20	N	P2O5	K20	N	P2O5	K20	N	P2O5	K20
	220	55	60	220	0	0						

Copies To:

Analysis Approved By:

Lori Scott, TLOS

Confidentiality Notice-This report is for the individual or Organization to whom it is addressed and contains confidential information. If received in error, please contact us immediately

Soil Test Report - Additional Information

- This report shall not be reproduced, except in full, without written approval of Laboratory Services
- **Bold** indicates analysis accredited to ISO/IEC 17025:2017 standard
- **[S]** Sample sent to an accredited lab for analysis
- **LA** indicates Lab Accident and the analysis was not able to be completed
- **LR** Lime Requirement
- The dates of performance of laboratory activity are available by contacting Laboratory Services at (902) 893-6565
- Result(s) apply to the sample as received, and relate only to the sample(s) tested

Conversions:

- 1 kg/ha = 0.89 lb/ac
- 1 tonne/ha = 0.45 ton/ac = 10 kg/100m^2
- 1 kg/ha/100 = 1 kg/100m^2
- to convert kg/ha to ppm divide by 2

Ratings:

- L = Low M = Medium H = High E = Excessive

Method References for Accredited Methods

- LSAL408 Determination of Mehlich III Extractable Major and Trace Metal Ions in Soil by ICP-OES (Modified Mehlich III, Commun. Soil. Sci. Plant Anal. 15(12): 1409- 1416)
- LSAL409 Determination of Soil Water pH and Lime Requirement using Adams-Evans Buffer by AS3010D pH Analyzer

The following Soil Related Factsheets are availabe on our website:
https://novascotia.ca/agriculture-labs

- How to take Field Soil Samples
- Understanding the Soils Test Report
