# Supplementary material for: Field evaluation of ascorbic acid seed priming on growth, physiology, yield, and nutritional quality of broccoli (Brassica oleracea L. var. italica ‘Gypsy’)
Source: Front Plant Sci. 2026 Sep 16;17:1897446. doi: 10.3389/fpls.2026.1897446 (PMC13624865; doi:10.3389/fpls.2026.1897446)
Supplement: Supplementary file 3 [file DataSheet5.pdf]

Tissue Report

Department of Agriculture
Laboratory Services
PO Box 890
Harlow Institute
Truro, NS B2N 5G6

novascotia.ca/agriculture-labs
Tel: 902-893-6565
Fax: 902-893-4193

DEVRIES, SJOERD
VRIESLAND FARM LTD
2666 HWY 289
MIDDLE STEWIACKE, NS
B0N 1C0

Client ID: C10556
Order ID: 2405528
Samples Reported: 5-Sep-24
Samples Received: 21-Aug-24
# of Samples Received: 20

Lab #	2405528-001		2405528-002		2405528-003		2405528-004	
Client Sample ID	DC1		DC2		DC3		DC4	
Registration #								
Sample Type	Broccoli		Broccoli		Broccoli		Broccoli	
Parameter	Result	Reporting Limit	Result	Reporting Limit	Result	Reporting Limit	Result	Reporting Limit
**Nitrogen (%)**	5.15	0.02	4.80	0.02	5.18	0.02	5.26	0.02
Calcium (%)	0.369	0.002	0.461	0.002	0.364	0.002	0.379	0.002
Potassium (%)	3.386	0.015	3.321	0.015	3.461	0.015	3.369	0.015
Magnesium (%)	0.218	0.002	0.233	0.002	0.246	0.002	0.231	0.002
Phosphorus (%)	0.729	0.001	0.715	0.001	0.758	0.001	0.703	0.001
Sodium (%)	0.030	0.015	0.027	0.015	0.034	0.015	0.040	0.015
Boron (ppm)	31.35	10.00	26.62	10.00	34.34	10.00	30.28	10.00
Copper (ppm)	ND	5.00	ND	5.00	ND	5.00	ND	5.00
Iron (ppm)	63.60	5.00	60.94	5.00	66.35	5.00	68.93	5.00
Manganese (ppm)	28.08	10.00	32.86	10.00	28.94	10.00	31.74	10.00
Zinc (ppm)	49.79	2.00	54.47	2.00	51.81	2.00	52.77	2.00

**Copies To:**	**Analysis Approved By:**
	Amy Sangster, Lab Manager

Confidentiality Notice-This report is for the individual or Organization to whom it is addressed and contains confidential information. If received in error, please contact us immediately

Tissue Report

Department of Agriculture
Laboratory Services
PO Box 890
Harlow Institute
Truro, NS B2N 5G6

novascotia.ca/agriculture-labs
Tel: 902-893-6565
Fax: 902-893-4193

DEVRIES, SJOERD
VRIESLAND FARM LTD
2666 HWY 289
MIDDLE STEWIACKE, NS
B0N 1C0

Client ID: C10556
Order ID: 2405528
Samples Reported: 5-Sep-24
Samples Received: 21-Aug-24
\# of Samples Received: 20

Lab #	2405528-005		2405528-006		2405528-007		2405528-008	
Client Sample ID	DC5		D01		D02		D03	
Registration #								
Sample Type	Broccoli		Broccoli		Broccoli		Broccoli	
Parameter	Result	Reporting Limit	Result	Reporting Limit	Result	Reporting Limit	Result	Reporting Limit
**Nitrogen (%)**	5.11	0.02	5.34	0.02	4.96	0.02	5.82	0.02
Calcium (%)	0.400	0.002	0.407	0.002	0.508	0.002	0.332	0.002
Potassium (%)	3.504	0.015	2.989	0.015	2.951	0.015	3.052	0.015
Magnesium (%)	0.232	0.002	0.224	0.002	0.249	0.002	0.231	0.002
Phosphorus (%)	0.779	0.001	0.669	0.001	0.685	0.001	0.745	0.001
Sodium (%)	0.028	0.015	0.031	0.015	0.034	0.015	0.041	0.015
Boron (ppm)	37.83	10.00	27.01	10.00	25.33	10.00	35.79	10.00
Copper (ppm)	ND	5.00	ND	5.00	ND	5.00	ND	5.00
Iron (ppm)	70.21	5.00	66.70	5.00	62.03	5.00	73.04	5.00
Manganese (ppm)	30.01	10.00	32.02	10.00	34.32	10.00	29.24	10.00
Zinc (ppm)	53.42	2.00	55.61	2.00	54.69	2.00	57.09	2.00

**Copies To:**	**Analysis Approved By:**
	Amy Sangster, Lab Manager

Confidentiality Notice-This report is for the individual or Organization to whom it is addressed and contains confidential information. If received in error, please contact us immediately

Tissue Report

Department of Agriculture
Laboratory Services
PO Box 890
Harlow Institute
Truro, NS B2N 5G6

novascotia.ca/agriculture-labs
Tel: 902-893-6565
Fax: 902-893-4193

DEVRIES, SJOERD
VRIESLAND FARM LTD
2666 HWY 289
MIDDLE STEWIACKE, NS
B0N 1C0

Client ID: C10556
Order ID: 2405528
Samples Reported: 5-Sep-24
Samples Received: 21-Aug-24
# of Samples Received: 20

Lab #	2405528-009		2405528-010		2405528-011		2405528-012	
Client Sample ID	D04		D05		D11		D12	
Registration #								
Sample Type	Broccoli		Broccoli		Broccoli		Broccoli	
Parameter	Result	Reporting Limit	Result	Reporting Limit	Result	Reporting Limit	Result	Reporting Limit
**Nitrogen (%)**	5.25	0.02	4.99	0.02	4.93	0.02	5.62	0.02
Calcium (%)	0.364	0.002	0.393	0.002	0.400	0.002	0.384	0.002
Potassium (%)	3.121	0.015	3.031	0.015	3.091	0.015	3.232	0.015
Magnesium (%)	0.204	0.002	0.220	0.002	0.213	0.002	0.224	0.002
Phosphorus (%)	0.727	0.001	0.672	0.001	0.683	0.001	0.758	0.001
Sodium (%)	0.028	0.015	0.025	0.015	0.029	0.015	0.023	0.015
Boron (ppm)	36.13	10.00	31.39	10.00	28.48	10.00	38.78	10.00
Copper (ppm)	ND	5.00	ND	5.00	ND	5.00	5.59	5.00
Iron (ppm)	68.56	5.00	60.22	5.00	60.62	5.00	71.38	5.00
Manganese (ppm)	29.64	10.00	27.84	10.00	30.29	10.00	29.33	10.00
Zinc (ppm)	53.18	2.00	46.24	2.00	49.89	2.00	52.14	2.00

Copies To:	Analysis Approved By:
	Amy Sangster, Lab Manager

Confidentiality Notice-This report is for the individual or Organization to whom it is addressed and contains confidential information. If received in error, please contact us immediately

Tissue Report

Department of Agriculture
Laboratory Services
PO Box 890
Harlow Institute
Truro, NS B2N 5G6

novascotia.ca/agriculture-labs
Tel: 902-893-6565
Fax: 902-893-4193

Client ID: C10556
Order ID: 2405528
Samples Reported: 5-Sep-24
Samples Received: 21-Aug-24
# of Samples Received: 20

DEVRIES, SJOERD
VRIESLAND FARM LTD
2666 HWY 289
MIDDLE STEWIACKE, NS
B0N 1C0

Lab #	2405528-013		2405528-014		2405528-015		2405528-016	
Client Sample ID	D13		D14		D15		D101	
Registration #								
Sample Type	Broccoli		Broccoli		Broccoli		Broccoli	
Parameter	Result	Reporting Limit	Result	Reporting Limit	Result	Reporting Limit	Result	Reporting Limit
**Nitrogen (%)**	5.58	0.02	5.30	0.02	5.38	0.02	4.79	0.02
Calcium (%)	0.368	0.002	0.307	0.002	0.526	0.002	0.438	0.002
Potassium (%)	3.136	0.015	3.108	0.015	2.963	0.015	2.992	0.015
Magnesium (%)	0.227	0.002	0.210	0.002	0.232	0.002	0.223	0.002
Phosphorus (%)	0.726	0.001	0.686	0.001	0.717	0.001	0.655	0.001
Sodium (%)	0.035	0.015	0.026	0.015	0.027	0.015	0.023	0.015
Boron (ppm)	34.04	10.00	33.11	10.00	26.48	10.00	26.97	10.00
Copper (ppm)	5.18	5.00	ND	5.00	ND	5.00	ND	5.00
Iron (ppm)	73.86	5.00	59.80	5.00	67.76	5.00	61.28	5.00
Manganese (ppm)	31.15	10.00	26.91	10.00	33.59	10.00	31.15	10.00
Zinc (ppm)	54.76	2.00	51.00	2.00	53.58	2.00	52.60	2.00

**Copies To:**	**Analysis Approved By:**
	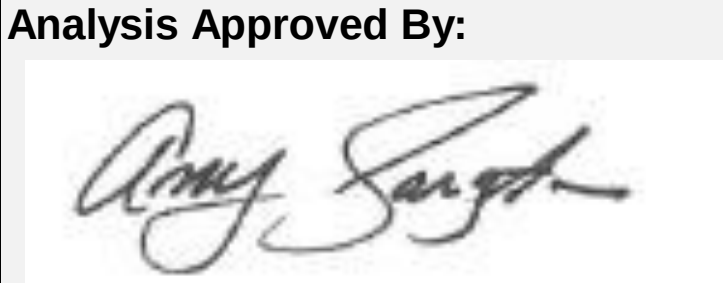 Amy Sangster, Lab Manager

Confidentiality Notice-This report is for the individual or Organization to whom it is addressed and contains confidential information. If received in error, please contact us immediately

Tissue Report

Department of Agriculture
Laboratory Services
PO Box 890
Harlow Institute
Truro, NS B2N 5G6

novascotia.ca/agriculture-labs
Tel: 902-893-6565
Fax: 902-893-4193

Client ID: C10556
Order ID: 2405528
Samples Reported: 5-Sep-24
Samples Received: 21-Aug-24
# of Samples Received: 20

DEVRIES, SJOERD
VRIESLAND FARM LTD
2666 HWY 289
MIDDLE STEWIACKE, NS
B0N 1C0

Lab #	2405528-017		2405528-018		2405528-019		2405528-020	
Client Sample ID	D102		D103		D104		D105	
Registration #								
Sample Type	Broccoli		Broccoli		Broccoli		Broccoli	
Parameter	Result	Reporting Limit	Result	Reporting Limit	Result	Reporting Limit	Result	Reporting Limit
**Nitrogen (%)**	5.34	0.02	4.58	0.02	4.77	0.02	4.93	0.02
Calcium (%)	0.416	0.002	0.417	0.002	0.317	0.002	0.357	0.002
Potassium (%)	2.852	0.015	3.017	0.015	3.211	0.015	3.005	0.015
Magnesium (%)	0.222	0.002	0.212	0.002	0.212	0.002	0.219	0.002
Phosphorus (%)	0.660	0.001	0.643	0.001	0.658	0.001	0.639	0.001
Sodium (%)	0.046	0.015	0.031	0.015	0.026	0.015	0.033	0.015
Boron (ppm)	26.65	10.00	29.16	10.00	29.90	10.00	28.12	10.00
Copper (ppm)	ND	5.00	ND	5.00	ND	5.00	ND	5.00
Iron (ppm)	67.80	5.00	72.92	5.00	60.95	5.00	63.08	5.00
Manganese (ppm)	31.61	10.00	31.02	10.00	29.40	10.00	32.58	10.00
Zinc (ppm)	59.07	2.00	50.70	2.00	50.03	2.00	54.33	2.00

Copies To:	Analysis Approved By:
	Amy Sangster, Lab Manager

Confidentiality Notice-This report is for the individual or Organization to whom it is addressed and contains confidential information. If received in error, please contact us immediately

Additional Information

- This report shall not be reproduced, except in full, without written approval of Laboratory Services
- **Bold** indicates analysis accredited to ISO/IEC 17025:2017 standard
- **[S]** Sample sent to an accredited lab for analysis
- **LA** indicates Lab Accident and the analysis was not able to be completed
- **ND** indicates the analysis value is below **reporting limit** which is the lowest conentration that will be reported for a specific method
- Result(s) apply to the sample as received, and relate only to the sample(s) tested
- The dates of laboratory activity are available by contacting Laboratory Services at (902) 893-6565

Method References for Accredited Methods

- LSAL410 Crude Protein / Nitrogen in Animal Feeds and Plant Tissue by Combustion Analysis

The following Plant Tissue Related Factsheets are availabe on our website:
http://novascotia.ca/agriculture-labs

- How to take a Plant Tissue Test
- Fruit Crop Tissue Sampling Guide
- Field Crop Tissue Sampling Guide
- Vegetable Crop Tissue Sampling Guide
- Understanding the Tissue Analysis Report
