# Supplementary material for: Field evaluation of ascorbic acid seed priming on growth, physiology, yield, and nutritional quality of broccoli (Brassica oleracea L. var. italica ‘Gypsy’)
Source: Front Plant Sci. 2026 Sep 16;17:1897446. doi: 10.3389/fpls.2026.1897446 (PMC13624865; doi:10.3389/fpls.2026.1897446)
Supplement: Supplementary file 4 [file DataSheet6.pdf]

Tissue Report

Department of Agriculture
Laboratory Services
PO Box 890
Harlow Institute
Truro, NS B2N 5G6

novascotia.ca/agriculture-labs
Tel: 902-893-6565
Fax: 902-893-4193

DEVRIES, SJOERD
VRIESLAND FARM LTD
2666 HWY 289
MIDDLE STEWIACKE, NS
B0N 1C0

Client ID: C10556
Order ID: 2405529
Samples Reported: 5-Sep-24
Samples Received: 21-Aug-24
# of Samples Received: 18

Lab #	2405529-001		2405529-002		2405529-003		2405529-004	
Client Sample ID	CC1		CC2		CC3		C01	
Registration #								
Sample Type	Broccoli		Broccoli		Broccoli		Broccoli	
Parameter	Result	Reporting Limit	Result	Reporting Limit	Result	Reporting Limit	Result	Reporting Limit
**Nitrogen (%)**	4.76	0.02	4.82	0.02	4.63	0.02	4.99	0.02
Calcium (%)	0.335	0.002	0.316	0.002	0.381	0.002	0.354	0.002
Potassium (%)	3.118	0.015	3.201	0.015	3.089	0.015	3.050	0.015
Magnesium (%)	0.206	0.002	0.189	0.002	0.193	0.002	0.183	0.002
Phosphorus (%)	0.668	0.001	0.705	0.001	0.687	0.001	0.700	0.001
Sodium (%)	0.024	0.015	0.036	0.015	0.031	0.015	0.027	0.015
Boron (ppm)	23.89	10.00	28.18	10.00	23.46	10.00	28.06	10.00
Copper (ppm)	ND	5.00	ND	5.00	ND	5.00	ND	5.00
Iron (ppm)	56.87	5.00	65.03	5.00	63.98	5.00	63.98	5.00
Manganese (ppm)	28.03	10.00	28.47	10.00	30.29	10.00	28.06	10.00
Zinc (ppm)	45.55	2.00	48.48	2.00	48.36	2.00	43.72	2.00

Copies To:

Analysis Approved By:

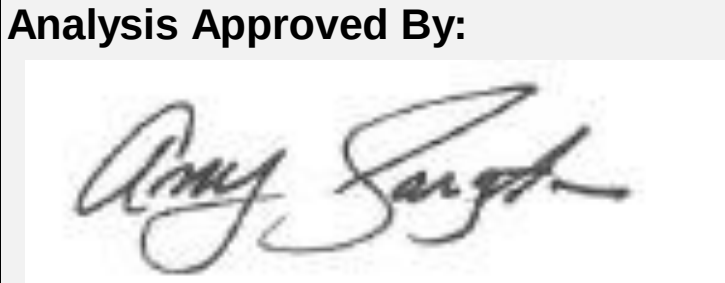

Amy Sangster, Lab Manager

Confidentiality Notice-This report is for the individual or Organization to whom it is addressed and contains confidential information. If received in error, please contact us immediately

Tissue Report

NOVA SCOTIA
Department of Agriculture
Laboratory Services
PO Box 890
Harlow Institute
Truro, NS B2N 5G6

novascotia.ca/agriculture-labs
Tel: 902-893-6565
Fax: 902-893-4193

Client ID: C10556
Order ID: 2405529
Samples Reported: 5-Sep-24
Samples Received: 21-Aug-24
# of Samples Received: 18

DEVRIES, SJOERD
VRIESLAND FARM LTD
2666 HWY 289
MIDDLE STEWIACKE, NS
B0N 1C0

Lab #	2405529-005		2405529-006		2405529-007		2405529-008	
Client Sample ID	C02		C03		C04		C05	
Registration #								
Sample Type	Broccoli		Broccoli		Broccoli		Broccoli	
Parameter	Result	Reporting Limit	Result	Reporting Limit	Result	Reporting Limit	Result	Reporting Limit
**Nitrogen (%)**	5.03	0.02	4.35	0.02	4.48	0.02	4.81	0.02
Calcium (%)	0.331	0.002	0.353	0.002	0.332	0.002	0.420	0.002
Potassium (%)	3.205	0.015	3.181	0.015	3.247	0.015	2.969	0.015
Magnesium (%)	0.209	0.002	0.185	0.002	0.172	0.002	0.168	0.002
Phosphorus (%)	0.732	0.001	0.641	0.001	0.637	0.001	0.691	0.001
Sodium (%)	0.034	0.015	0.026	0.015	0.033	0.015	0.046	0.015
Boron (ppm)	29.70	10.00	26.66	10.00	23.77	10.00	24.55	10.00
Copper (ppm)	ND	5.00	ND	5.00	ND	5.00	ND	5.00
Iron (ppm)	65.49	5.00	53.88	5.00	51.11	5.00	63.27	5.00
Manganese (ppm)	29.80	10.00	27.58	10.00	25.84	10.00	27.15	10.00
Zinc (ppm)	54.43	2.00	40.97	2.00	41.09	2.00	44.47	2.00

Copies To:	Analysis Approved By:
	Amy Sangster, Lab Manager

Confidentiality Notice-This report is for the individual or Organization to whom it is addressed and contains confidential information. If received in error, please contact us immediately

Tissue Report

Department of Agriculture
Laboratory Services
PO Box 890
Harlow Institute
Truro, NS B2N 5G6

novascotia.ca/agriculture-labs
Tel: 902-893-6565
Fax: 902-893-4193

Client ID: C10556
Order ID: 2405529
Samples Reported: 5-Sep-24
Samples Received: 21-Aug-24
# of Samples Received: 18

DEVRIES, SJOERD
VRIESLAND FARM LTD
2666 HWY 289
MIDDLE STEWIACKE, NS
B0N 1C0

Lab #	2405529-009		2405529-010		2405529-011		2405529-012	
Client Sample ID	C11		C12		C13		C14	
Registration #								
Sample Type	Broccoli		Broccoli		Broccoli		Broccoli	
Parameter	Result	Reporting Limit	Result	Reporting Limit	Result	Reporting Limit	Result	Reporting Limit
**Nitrogen (%)**	4.48	0.02	4.66	0.02	4.72	0.02	4.47	0.02
Calcium (%)	0.355	0.002	0.297	0.002	0.343	0.002	0.324	0.002
Potassium (%)	3.146	0.015	3.042	0.015	3.066	0.015	3.137	0.015
Magnesium (%)	0.181	0.002	0.176	0.002	0.172	0.002	0.198	0.002
Phosphorus (%)	0.691	0.001	0.664	0.001	0.661	0.001	0.656	0.001
Sodium (%)	0.028	0.015	0.031	0.015	0.029	0.015	0.024	0.015
Boron (ppm)	26.14	10.00	27.72	10.00	27.51	10.00	23.27	10.00
Copper (ppm)	ND	5.00	ND	5.00	ND	5.00	ND	5.00
Iron (ppm)	59.39	5.00	56.39	5.00	58.55	5.00	55.52	5.00
Manganese (ppm)	27.86	10.00	27.30	10.00	26.79	10.00	29.57	10.00
Zinc (ppm)	44.42	2.00	42.10	2.00	44.34	2.00	45.50	2.00

Copies To:	Analysis Approved By:
	Amy Sangster, Lab Manager

Confidentiality Notice-This report is for the individual or Organization to whom it is addressed and contains confidential information. If received in error, please contact us immediately

Tissue Report

Department of Agriculture
Laboratory Services
PO Box 890
Harlow Institute
Truro, NS B2N 5G6

novascotia.ca/agriculture-labs
Tel: 902-893-6565
Fax: 902-893-4193

Client ID: C10556
Order ID: 2405529
Samples Reported: 5-Sep-24
Samples Received: 21-Aug-24
# of Samples Received: 18

DEVRIES, SJOERD
VRIESLAND FARM LTD
2666 HWY 289
MIDDLE STEWIACKE, NS
B0N 1C0

Lab #	2405529-013		2405529-014		2405529-015		2405529-016	
Client Sample ID	C15		C101		C102		C103	
Registration #								
Sample Type	Broccoli		Broccoli		Broccoli		Broccoli	
Parameter	Result	Reporting Limit	Result	Reporting Limit	Result	Reporting Limit	Result	Reporting Limit
**Nitrogen (%)**	4.58	0.02	4.64	0.02	4.98	0.02	4.68	0.02
Calcium (%)	0.440	0.002	0.346	0.002	0.359	0.002	0.381	0.002
Potassium (%)	3.082	0.015	2.956	0.015	3.194	0.015	3.112	0.015
Magnesium (%)	0.191	0.002	0.176	0.002	0.206	0.002	0.187	0.002
Phosphorus (%)	0.678	0.001	0.666	0.001	0.709	0.001	0.694	0.001
Sodium (%)	0.039	0.015	0.039	0.015	0.023	0.015	0.035	0.015
Boron (ppm)	29.53	10.00	21.90	10.00	32.42	10.00	29.62	10.00
Copper (ppm)	ND	5.00	ND	5.00	ND	5.00	ND	5.00
Iron (ppm)	57.73	5.00	60.83	5.00	64.58	5.00	59.11	5.00
Manganese (ppm)	31.84	10.00	26.72	10.00	29.76	10.00	31.37	10.00
Zinc (ppm)	43.37	2.00	47.34	2.00	48.94	2.00	45.36	2.00

Copies To:	Analysis Approved By:
	Amy Sangster, Lab Manager

Confidentiality Notice-This report is for the individual or Organization to whom it is addressed and contains confidential information. If received in error, please contact us immediately

Tissue Report

Department of Agriculture
Laboratory Services
PO Box 890
Harlow Institute
Truro, NS B2N 5G6

novascotia.ca/agriculture-labs
Tel: 902-893-6565
Fax: 902-893-4193

DEVRIES, SJOERD
VRIESLAND FARM LTD
2666 HWY 289
MIDDLE STEWIACKE, NS
B0N 1C0

Client ID: C10556
Order ID: 2405529
Samples Reported: 5-Sep-24
Samples Received: 21-Aug-24
# of Samples Received: 18

Lab #	2405529-017		2405529-018					
Client Sample ID	C104		C105					
Registration #								
Sample Type	Broccoli		Broccoli					
Parameter	Result	Reporting Limit	Result	Reporting Limit	Result	Reporting Limit	Result	Reporting Limit
**Nitrogen (%)**	4.61	0.02	4.30	0.02				
Calcium (%)	0.374	0.002	0.276	0.002				
Potassium (%)	3.147	0.015	3.176	0.015				
Magnesium (%)	0.203	0.002	0.177	0.002				
Phosphorus (%)	0.686	0.001	0.660	0.001				
Sodium (%)	0.025	0.015	0.025	0.015				
Boron (ppm)	26.26	10.00	31.14	10.00				
Copper (ppm)	ND	5.00	ND	5.00				
Iron (ppm)	62.91	5.00	56.89	5.00				
Manganese (ppm)	31.08	10.00	25.93	10.00				
Zinc (ppm)	45.34	2.00	39.29	2.00				

Copies To:

Analysis Approved By:

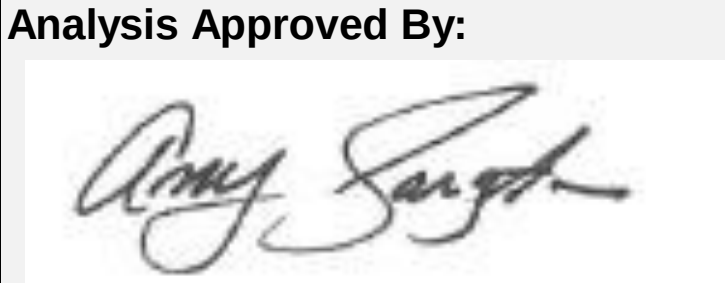

Amy Sangster, Lab Manager

Confidentiality Notice-This report is for the individual or Organization to whom it is addressed and contains confidential information. If received in error, please contact us immediately

Additional Information

- This report shall not be reproduced, except in full, without written approval of Laboratory Services
- **Bold** indicates analysis accredited to ISO/IEC 17025:2017 standard
- **[S]** Sample sent to an accredited lab for analysis
- **LA** indicates Lab Accident and the analysis was not able to be completed
- **ND** indicates the analysis value is below **reporting limit** which is the lowest conentration that will be reported for a specific method
- Result(s) apply to the sample as received, and relate only to the sample(s) tested
- The dates of laboratory activity are available by contacting Laboratory Services at (902) 893-6565

Method References for Accredited Methods

- LSAL410 Crude Protein / Nitrogen in Animal Feeds and Plant Tissue by Combustion Analysis

The following Plant Tissue Related Factsheets are availabe on our website:
http://novascotia.ca/agriculture-labs

- How to take a Plant Tissue Test
- Fruit Crop Tissue Sampling Guide
- Field Crop Tissue Sampling Guide
- Vegetable Crop Tissue Sampling Guide
- Understanding the Tissue Analysis Report
