# Supplementary material for: Field evaluation of ascorbic acid seed priming on growth, physiology, yield, and nutritional quality of broccoli (Brassica oleracea L. var. italica ‘Gypsy’)
Source: Front Plant Sci. 2026 Sep 16;17:1897446. doi: 10.3389/fpls.2026.1897446 (PMC13624865; doi:10.3389/fpls.2026.1897446)
Supplement: Supplementary file 5 [file DataSheet7.pdf]

Tissue Report

Department of Agriculture
Laboratory Services
PO Box 890
Harlow Institute
Truro, NS B2N 5G6

novascotia.ca/agriculture-labs
Tel: 902-893-6565
Fax: 902-893-4193

Client ID: C10556
Order ID: 2506651
Samples Reported: 17-Oct-25
Samples Received: 9-Oct-25
# of Samples Received: 40

DEVRIES, SJOERD
VRIESLAND FARM LTD
2666 HWY 289
MIDDLE STEWIACKE, NS
B0N 1C0

Lab #	2506651-001		2506651-002		2506651-003		2506651-004	
Client Sample ID	DC 1		DC 2		DC 3		DC 4	
Registration #								
Sample Type	Broccoli		Broccoli		Broccoli		Broccoli	
Parameter	Result	Reporting Limit	Result	Reporting Limit	Result	Reporting Limit	Result	Reporting Limit
**Nitrogen (%)**	6.24	0.02	7.33	0.02	5.80	0.02	6.19	0.02
Calcium (%)	0.229	0.002	0.260	0.002	0.196	0.002	0.211	0.002
Potassium (%)	3.165	0.015	2.870	0.015	3.220	0.015	3.219	0.015
Magnesium (%)	0.206	0.002	0.193	0.002	0.208	0.002	0.203	0.002
Phosphorus (%)	0.801	0.001	0.810	0.001	0.775	0.001	0.792	0.001
Sodium (%)	0.058	0.015	0.054	0.015	0.053	0.015	0.048	0.015
Boron (ppm)	18.08	10.00	18.86	10.00	19.22	10.00	17.46	10.00
Copper (ppm)	6.26	5.00	6.84	5.00	6.09	5.00	6.17	5.00
Iron (ppm)	114.31	5.00	86.85	5.00	71.21	5.00	68.14	5.00
Manganese (ppm)	23.06	10.00	25.59	10.00	23.52	10.00	21.43	10.00
Zinc (ppm)	237.96	2.00	99.38	2.00	78.13	2.00	71.54	2.00

**Copies To:**	**Analysis Approved By:**
	L. Scott<br>Lori Scott, TLOS

Confidentiality Notice-This report is for the individual or Organization to whom it is addressed and contains confidential information. If received in error, please contact us immediately

Tissue Report

Department of Agriculture
Laboratory Services
PO Box 890
Harlow Institute
Truro, NS B2N 5G6

novascotia.ca/agriculture-labs
Tel: 902-893-6565
Fax: 902-893-4193

Client ID: C10556
Order ID: 2506651
Samples Reported: 17-Oct-25
Samples Received: 9-Oct-25
# of Samples Received: 40

DEVRIES, SJOERD
VRIESLAND FARM LTD
2666 HWY 289
MIDDLE STEWIACKE, NS
B0N 1C0

Lab #	2506651-005		2506651-006		2506651-007		2506651-008	
Client Sample ID	DC 5		D0 1		D0 2		D0 3	
Registration #								
Sample Type	Broccoli		Broccoli		Broccoli		Broccoli	
Parameter	Result	Reporting Limit	Result	Reporting Limit	Result	Reporting Limit	Result	Reporting Limit
**Nitrogen (%)**	7.24	0.02	6.93	0.02	6.07	0.02	6.86	0.02
Calcium (%)	0.277	0.002	0.207	0.002	0.196	0.002	0.262	0.002
Potassium (%)	3.053	0.015	3.033	0.015	3.276	0.015	2.984	0.015
Magnesium (%)	0.215	0.002	0.214	0.002	0.206	0.002	0.201	0.002
Phosphorus (%)	0.891	0.001	0.817	0.001	0.818	0.001	0.754	0.001
Sodium (%)	0.034	0.015	0.038	0.015	0.046	0.015	0.045	0.015
Boron (ppm)	25.50	10.00	21.73	10.00	17.66	10.00	19.85	10.00
Copper (ppm)	7.02	5.00	6.73	5.00	5.99	5.00	6.44	5.00
Iron (ppm)	83.43	5.00	79.66	5.00	72.91	5.00	76.85	5.00
Manganese (ppm)	23.20	10.00	23.77	10.00	23.29	10.00	24.54	10.00
Zinc (ppm)	83.52	2.00	77.35	2.00	70.39	2.00	84.10	2.00

Copies To:	Analysis Approved By:
	L. Scott.<br>Lori Scott, TLOS

Confidentiality Notice-This report is for the individual or Organization to whom it is addressed and contains confidential information. If received in error, please contact us immediately

Tissue Report

Department of Agriculture
Laboratory Services
PO Box 890
Harlow Institute
Truro, NS B2N 5G6

novascotia.ca/agriculture-labs
Tel: 902-893-6565
Fax: 902-893-4193

DEVRIES, SJOERD
VRIESLAND FARM LTD
2666 HWY 289
MIDDLE STEWIACKE, NS
B0N 1C0

Client ID: C10556
Order ID: 2506651
Samples Reported: 17-Oct-25
Samples Received: 9-Oct-25
# of Samples Received: 40

Lab #	2506651-009		2506651-010		2506651-011		2506651-012	
Client Sample ID	D0 4		DO 5		D 11		D 12	
Registration #								
Sample Type	Broccoli		Broccoli		Broccoli		Broccoli	
Parameter	Result	Reporting Limit	Result	Reporting Limit	Result	Reporting Limit	Result	Reporting Limit
**Nitrogen (%)**	6.35	0.02	6.89	0.02	6.41	0.02	6.19	0.02
Calcium (%)	0.248	0.002	0.282	0.002	0.238	0.002	0.228	0.002
Potassium (%)	3.408	0.015	3.173	0.015	3.345	0.015	3.134	0.015
Magnesium (%)	0.221	0.002	0.212	0.002	0.228	0.002	0.199	0.002
Phosphorus (%)	0.831	0.001	0.861	0.001	0.868	0.001	0.819	0.001
Sodium (%)	0.043	0.015	0.048	0.015	0.054	0.015	0.046	0.015
Boron (ppm)	24.48	10.00	22.13	10.00	27.10	10.00	20.78	10.00
Copper (ppm)	6.91	5.00	7.15	5.00	6.65	5.00	6.44	5.00
Iron (ppm)	74.36	5.00	104.00	5.00	77.75	5.00	93.12	5.00
Manganese (ppm)	25.22	10.00	63.63	10.00	25.54	10.00	23.25	10.00
Zinc (ppm)	74.91	2.00	78.03	2.00	79.13	2.00	73.49	2.00

**Copies To:**	**Analysis Approved By:**
	L. Scott.<br>Lori Scott, TLOS

Confidentiality Notice-This report is for the individual or Organization to whom it is addressed and contains confidential information. If received in error, please contact us immediately

Tissue Report

Department of Agriculture
Laboratory Services
PO Box 890
Harlow Institute
Truro, NS B2N 5G6

novascotia.ca/agriculture-labs
Tel: 902-893-6565
Fax: 902-893-4193

DEVRIES, SJOERD
VRIESLAND FARM LTD
2666 HWY 289
MIDDLE STEWIACKE, NS
B0N 1C0

Client ID: C10556
Order ID: 2506651
Samples Reported: 17-Oct-25
Samples Received: 9-Oct-25
# of Samples Received: 40

Lab #	2506651-013		2506651-014		2506651-015		2506651-016	
Client Sample ID	D 13		D 14		D 15		D 101	
Registration #								
Sample Type	Broccoli		Broccoli		Broccoli		Broccoli	
Parameter	Result	Reporting Limit	Result	Reporting Limit	Result	Reporting Limit	Result	Reporting Limit
**Nitrogen (%)**	6.22	0.02	5.95	0.02	5.91	0.02	5.50	0.02
Calcium (%)	0.274	0.002	0.209	0.002	0.223	0.002	0.224	0.002
Potassium (%)	3.547	0.015	3.360	0.015	3.245	0.015	3.152	0.015
Magnesium (%)	0.235	0.002	0.214	0.002	0.201	0.002	0.216	0.002
Phosphorus (%)	0.809	0.001	0.820	0.001	0.790	0.001	0.732	0.001
Sodium (%)	0.062	0.015	0.053	0.015	0.056	0.015	0.046	0.015
Boron (ppm)	23.09	10.00	19.67	10.00	18.47	10.00	18.15	10.00
Copper (ppm)	7.20	5.00	7.06	5.00	5.94	5.00	6.31	5.00
Iron (ppm)	80.16	5.00	74.02	5.00	68.09	5.00	58.29	5.00
Manganese (ppm)	28.96	10.00	23.54	10.00	22.32	10.00	22.87	10.00
Zinc (ppm)	91.03	2.00	72.37	2.00	63.17	2.00	60.15	2.00

**Copies To:**	**Analysis Approved By:**
	L. Scott. Lori Scott, TLOS

Confidentiality Notice-This report is for the individual or Organization to whom it is addressed and contains confidential information. If received in error, please contact us immediately

Tissue Report

Department of Agriculture
Laboratory Services
PO Box 890
Harlow Institute
Truro, NS B2N 5G6

novascotia.ca/agriculture-labs
Tel: 902-893-6565
Fax: 902-893-4193

Client ID: C10556
Order ID: 2506651
Samples Reported: 17-Oct-25
Samples Received: 9-Oct-25
# of Samples Received: 40

DEVRIES, SJOERD
VRIESLAND FARM LTD
2666 HWY 289
MIDDLE STEWIACKE, NS
B0N 1C0

Lab #	2506651-017		2506651-018		2506651-019		2506651-020	
Client Sample ID	D 102		D 103		D 104		D 105	
Registration #								
Sample Type	Broccoli		Broccoli		Broccoli		Broccoli	
Parameter	Result	Reporting Limit	Result	Reporting Limit	Result	Reporting Limit	Result	Reporting Limit
**Nitrogen (%)**	6.12	0.02	6.02	0.02	7.22	0.02	6.01	0.02
Calcium (%)	0.207	0.002	0.228	0.002	0.266	0.002	0.252	0.002
Potassium (%)	3.232	0.015	3.244	0.015	3.071	0.015	3.221	0.015
Magnesium (%)	0.217	0.002	0.198	0.002	0.214	0.002	0.201	0.002
Phosphorus (%)	0.764	0.001	0.816	0.001	0.793	0.001	0.742	0.001
Sodium (%)	0.047	0.015	0.047	0.015	0.047	0.015	0.057	0.015
Boron (ppm)	19.18	10.00	20.91	10.00	23.10	10.00	20.52	10.00
Copper (ppm)	6.51	5.00	6.47	5.00	7.14	5.00	5.59	5.00
Iron (ppm)	72.12	5.00	73.91	5.00	80.60	5.00	67.19	5.00
Manganese (ppm)	23.31	10.00	22.66	10.00	27.05	10.00	25.20	10.00
Zinc (ppm)	73.27	2.00	67.98	2.00	93.47	2.00	68.34	2.00

**Copies To:**	**Analysis Approved By:**
	L. Scott<br>Lori Scott, TLOS

Confidentiality Notice-This report is for the individual or Organization to whom it is addressed and contains confidential information. If received in error, please contact us immediately

Tissue Report

Department of Agriculture
Laboratory Services
PO Box 890
Harlow Institute
Truro, NS B2N 5G6

novascotia.ca/agriculture-labs
Tel: 902-893-6565
Fax: 902-893-4193

DEVRIES, SJOERD
VRIESLAND FARM LTD
2666 HWY 289
MIDDLE STEWIACKE, NS
B0N 1C0

Client ID: C10556
Order ID: 2506651
Samples Reported: 17-Oct-25
Samples Received: 9-Oct-25
# of Samples Received: 40

Lab #	2506651-021		2506651-022		2506651-023		2506651-024	
Client Sample ID	CC 1		CC 2		CC 3		CC 4	
Registration #								
Sample Type	Broccoli		Broccoli		Broccoli		Broccoli	
Parameter	Result	Reporting Limit	Result	Reporting Limit	Result	Reporting Limit	Result	Reporting Limit
**Nitrogen (%)**	6.27	0.02	6.38	0.02	7.43	0.02	7.00	0.02
Calcium (%)	0.269	0.002	0.261	0.002	0.306	0.002	0.279	0.002
Potassium (%)	3.246	0.015	3.169	0.015	3.055	0.015	3.229	0.015
Magnesium (%)	0.204	0.002	0.198	0.002	0.195	0.002	0.223	0.002
Phosphorus (%)	0.834	0.001	0.833	0.001	0.901	0.001	0.905	0.001
Sodium (%)	0.035	0.015	0.049	0.015	0.024	0.015	0.032	0.015
Boron (ppm)	19.82	10.00	19.03	10.00	27.91	10.00	26.79	10.00
Copper (ppm)	6.04	5.00	7.06	5.00	7.28	5.00	6.97	5.00
Iron (ppm)	72.77	5.00	80.23	5.00	80.36	5.00	82.83	5.00
Manganese (ppm)	25.37	10.00	25.16	10.00	24.58	10.00	25.30	10.00
Zinc (ppm)	71.30	2.00	77.21	2.00	77.36	2.00	75.95	2.00

**Copies To:**	**Analysis Approved By:**
	L. Scott. Lori Scott, TLOS

Confidentiality Notice-This report is for the individual or Organization to whom it is addressed and contains confidential information. If received in error, please contact us immediately

Tissue Report

Department of Agriculture
Laboratory Services
PO Box 890
Harlow Institute
Truro, NS B2N 5G6

novascotia.ca/agriculture-labs
Tel: 902-893-6565
Fax: 902-893-4193

Client ID: C10556
Order ID: 2506651
Samples Reported: 17-Oct-25
Samples Received: 9-Oct-25
# of Samples Received: 40

DEVRIES, SJOERD
VRIESLAND FARM LTD
2666 HWY 289
MIDDLE STEWIACKE, NS
B0N 1C0

Lab #	2506651-025		2506651-026		2506651-027		2506651-028	
Client Sample ID	CC 5		C0 1		CO 2		CO 3	
Registration #								
Sample Type	Broccoli		Broccoli		Broccoli		Broccoli	
Parameter	Result	Reporting Limit	Result	Reporting Limit	Result	Reporting Limit	Result	Reporting Limit
**Nitrogen (%)**	7.00	0.02	5.80	0.02	6.29	0.02	6.18	0.02
Calcium (%)	0.256	0.002	0.239	0.002	0.221	0.002	0.260	0.002
Potassium (%)	3.203	0.015	3.163	0.015	3.188	0.015	3.284	0.015
Magnesium (%)	0.186	0.002	0.194	0.002	0.199	0.002	0.209	0.002
Phosphorus (%)	0.848	0.001	0.774	0.001	0.790	0.001	0.826	0.001
Sodium (%)	0.042	0.015	0.049	0.015	0.039	0.015	0.037	0.015
Boron (ppm)	20.23	10.00	20.15	10.00	20.37	10.00	22.10	10.00
Copper (ppm)	6.79	5.00	6.62	5.00	5.98	5.00	7.04	5.00
Iron (ppm)	71.32	5.00	71.58	5.00	68.68	5.00	79.24	5.00
Manganese (ppm)	24.22	10.00	25.17	10.00	23.87	10.00	25.95	10.00
Zinc (ppm)	81.53	2.00	73.75	2.00	66.26	2.00	75.89	2.00

Copies To:	Analysis Approved By:
	L. Scott.<br>Lori Scott, TLOS

Confidentiality Notice-This report is for the individual or Organization to whom it is addressed and contains confidential information. If received in error, please contact us immediately

Tissue Report

Department of Agriculture
Laboratory Services
PO Box 890
Harlow Institute
Truro, NS B2N 5G6

novascotia.ca/agriculture-labs
Tel: 902-893-6565
Fax: 902-893-4193

DEVRIES, SJOERD
VRIESLAND FARM LTD
2666 HWY 289
MIDDLE STEWIACKE, NS
B0N 1C0

Client ID: C10556
Order ID: 2506651
Samples Reported: 17-Oct-25
Samples Received: 9-Oct-25
# of Samples Received: 40

Lab #	2506651-029		2506651-030		2506651-031		2506651-032	
Client Sample ID	CO 4		CO 5		C 11		C 12	
Registration #								
Sample Type	Broccoli		Broccoli		Broccoli		Broccoli	
Parameter	Result	Reporting Limit	Result	Reporting Limit	Result	Reporting Limit	Result	Reporting Limit
**Nitrogen (%)**	6.02	0.02	5.63	0.02	5.64	0.02	6.37	0.02
Calcium (%)	0.240	0.002	0.258	0.002	0.295	0.002	0.237	0.002
Potassium (%)	3.242	0.015	3.121	0.015	3.354	0.015	3.233	0.015
Magnesium (%)	0.201	0.002	0.176	0.002	0.239	0.002	0.193	0.002
Phosphorus (%)	0.813	0.001	0.756	0.001	0.822	0.001	0.834	0.001
Sodium (%)	0.035	0.015	0.041	0.015	0.039	0.015	0.032	0.015
Boron (ppm)	20.86	10.00	17.45	10.00	24.87	10.00	20.76	10.00
Copper (ppm)	5.79	5.00	5.88	5.00	7.03	5.00	6.37	5.00
Iron (ppm)	70.43	5.00	65.90	5.00	82.39	5.00	72.40	5.00
Manganese (ppm)	24.67	10.00	23.83	10.00	28.71	10.00	24.44	10.00
Zinc (ppm)	70.28	2.00	68.71	2.00	69.33	2.00	72.64	2.00

Copies To:	Analysis Approved By:
	L. Scott.<br>Lori Scott, TLOS

Confidentiality Notice-This report is for the individual or Organization to whom it is addressed and contains confidential information. If received in error, please contact us immediately

Tissue Report

Department of Agriculture
Laboratory Services
PO Box 890
Harlow Institute
Truro, NS B2N 5G6

novascotia.ca/agriculture-labs
Tel: 902-893-6565
Fax: 902-893-4193

Client ID: C10556
Order ID: 2506651
Samples Reported: 17-Oct-25
Samples Received: 9-Oct-25
# of Samples Received: 40

DEVRIES, SJOERD
VRIESLAND FARM LTD
2666 HWY 289
MIDDLE STEWIACKE, NS
B0N 1C0

Lab #	2506651-033		2506651-034		2506651-035		2506651-036	
Client Sample ID	C 13		C 14		C 15		C 101	
Registration #								
Sample Type	Broccoli		Broccoli		Broccoli		Broccoli	
Parameter	Result	Reporting Limit	Result	Reporting Limit	Result	Reporting Limit	Result	Reporting Limit
**Nitrogen (%)**	7.05	0.02	6.40	0.02	6.44	0.02	5.96	0.02
Calcium (%)	0.290	0.002	0.238	0.002	0.253	0.002	0.278	0.002
Potassium (%)	3.165	0.015	3.358	0.015	3.254	0.015	3.326	0.015
Magnesium (%)	0.214	0.002	0.193	0.002	0.195	0.002	0.222	0.002
Phosphorus (%)	0.863	0.001	0.870	0.001	0.860	0.001	0.850	0.001
Sodium (%)	0.024	0.015	0.036	0.015	0.038	0.015	0.052	0.015
Boron (ppm)	28.27	10.00	21.55	10.00	21.32	10.00	20.88	10.00
Copper (ppm)	6.76	5.00	6.34	5.00	6.78	5.00	6.80	5.00
Iron (ppm)	78.67	5.00	74.40	5.00	75.33	5.00	79.37	5.00
Manganese (ppm)	25.30	10.00	23.89	10.00	24.43	10.00	27.51	10.00
Zinc (ppm)	72.55	2.00	77.72	2.00	77.83	2.00	71.31	2.00

**Copies To:**	**Analysis Approved By:**
	L. Scott.<br>Lori Scott, TLOS

Confidentiality Notice-This report is for the individual or Organization to whom it is addressed and contains confidential information. If received in error, please contact us immediately

Tissue Report

Department of Agriculture
Laboratory Services
PO Box 890
Harlow Institute
Truro, NS B2N 5G6

novascotia.ca/agriculture-labs
Tel: 902-893-6565
Fax: 902-893-4193

DEVRIES, SJOERD
VRIESLAND FARM LTD
2666 HWY 289
MIDDLE STEWIACKE, NS
B0N 1C0

Client ID: C10556
Order ID: 2506651
Samples Reported: 17-Oct-25
Samples Received: 9-Oct-25
# of Samples Received: 40

Lab #	2506651-037		2506651-038		2506651-039		2506651-040	
Client Sample ID	C 102		C 103		C 104		C 105	
Registration #								
Sample Type	Broccoli		Broccoli		Broccoli		Broccoli	
Parameter	Result	Reporting Limit	Result	Reporting Limit	Result	Reporting Limit	Result	Reporting Limit
**Nitrogen (%)**	5.86	0.02	7.25	0.02	6.48	0.02	6.20	0.02
Calcium (%)	0.230	0.002	0.289	0.002	0.249	0.002	0.244	0.002
Potassium (%)	3.272	0.015	3.197	0.015	3.231	0.015	3.247	0.015
Magnesium (%)	0.193	0.002	0.211	0.002	0.218	0.002	0.198	0.002
Phosphorus (%)	0.795	0.001	0.911	0.001	0.882	0.001	0.826	0.001
Sodium (%)	0.035	0.015	0.029	0.015	0.039	0.015	0.036	0.015
Boron (ppm)	19.38	10.00	28.07	10.00	24.88	10.00	18.04	10.00
Copper (ppm)	6.06	5.00	7.29	5.00	6.70	5.00	8.49	5.00
Iron (ppm)	67.76	5.00	83.75	5.00	80.23	5.00	77.35	5.00
Manganese (ppm)	23.81	10.00	24.47	10.00	25.58	10.00	25.72	10.00
Zinc (ppm)	69.39	2.00	77.94	2.00	74.73	2.00	78.99	2.00

**Copies To:**	**Analysis Approved By:**
	L. Scott.<br>Lori Scott, TLOS

Confidentiality Notice-This report is for the individual or Organization to whom it is addressed and contains confidential information. If received in error, please contact us immediately

Additional Information

- This report shall not be reproduced, except in full, without written approval of Laboratory Services
- **Bold** indicates analysis accredited to ISO/IEC 17025:2017 standard
- **[S]** Sample sent to an accredited lab for analysis
- **LA** indicates Lab Accident and the analysis was not able to be completed
- **ND** indicates the analysis value is below **reporting limit** which is the lowest conentration that will be reported for a specific method
- Result(s) apply to the sample as received, and relate only to the sample(s) tested
- The dates of laboratory activity are available by contacting Laboratory Services at (902) 893-6565

Method References for Accredited Methods

- LSAL410 Crude Protein / Nitrogen in Animal Feeds and Plant Tissue by Combustion Analysis

The following Plant Tissue Related Factsheets are availabe on our website:
http://novascotia.ca/agriculture-labs

- How to take a Plant Tissue Test
- Fruit Crop Tissue Sampling Guide
- Field Crop Tissue Sampling Guide
- Vegetable Crop Tissue Sampling Guide
- Understanding the Tissue Analysis Report
