# Supplementary material for: Field evaluation of ascorbic acid seed priming on growth, physiology, yield, and nutritional quality of broccoli (Brassica oleracea L. var. italica ‘Gypsy’)
Source: Front Plant Sci. 2026 Sep 16;17:1897446. doi: 10.3389/fpls.2026.1897446 (PMC13624865; doi:10.3389/fpls.2026.1897446)
Supplement: Supplementary file 7 [file DataSheet9.pdf]

1 Supplementary Figures and Tables

1.1 Supplementary Tables

Supplementary table S1: Environmental conditions during gas-exchange sampling of broccoli plant

Date of sampling	PAR, Q (µmol m^{-2} s^{-1})	CO_2 concentration (ppm)	Chamber temperature (°C)	Leaf temperature (°C)
25 June 2024	693	419	24.3	23.6
2 July 2024	612	427	25.0	24.1
9 July 2024	721	441	25.2	22.7
16 July 2024	687	450	25.9	21.8
23 July 2024	680	462	25.7	23.8
30 July 2024	835	481	25.7	24.6
6 August 2024	684	438	26.8	25.4
16 September 2025	463	479	19.2	18.9
23 September 2025	494	401	18.4	18.1
1 October 2025	448	433	16.8	15.2
**Mean ± SD**	**631.7 ± 125.9**	**443.1 ± 25.5**	**23.3 ± 3.7**	**21.8 ± 3.3**

Supplementary Table S2: Comparison of covariance structures for repeated-measures analysis

Response variable	Compound symmetry (CS)	First-order autoregressive [AR (1)]	Unstructured (UN)	Selected covariance structure
**Plant height**	-78.5	-102.4	Did not converge	AR (1)
**Canopy Length**	533.7	522.0	Did not converge	AR (1)
**Stem girth**	-48.6	-88.0	Did not converge	AR (1)
**Leaf area**	2202.2	2128.1	2106.1	UN
**Net photosynthetic rate (Pn)**	225.7	220.0	221.7	AR (1)
**Stomatal conductance (gs)**	70.1	73.3	Did not converge	CS
**Transpiration rate (E)**	171.3	172.2	Did not converge	CS
**Chlorophyll content**	540.1	540.4	504.2	CS

Supplementary table S3: Repeated-measures ANOVA results for the effects of treatment, week, and their interaction on morpho-physiological and photosynthetic traits of broccoli plants.

Response variable	Covariance structure	Effect	DF	F value	P value
**Plant height**	AR(1)	Blocks	3	13.82	n.e.
		Trt	3	2.77	0.0468
		Week	6	71.47	<.0001
		Trt*Week	18	0.14	1.0000
**Canopy length**	AR(1)	Blocks	3	24.14	n.e.
		Trt	3	2.97	0.0366
		Week	6	52.37	<.0001
		Trt*Week	18	0.14	1.0000
**Stem girth**	AR(1)	Blocks	3	16.53	n.e.
		Trt	3	3.62	0.0165
		Week	6	50.65	<.0001
		Trt*Week	18	0.32	0.9954
**Leaf area**	UN	Blocks	3	53.90	n.e.
		Trt	3	3.96	0.0110
		Week	6	3.13	0.0083
		Trt*Week	18	0.91	0.5741

**Net photosynthetic rate**	AR(1)	Blocks	3	0.98	n.e.
		Trt	3	175.69	<.0001
		Week	6	4.41	0.0010
		Trt*Week	18	2.09	0.0181
**Transpiration rate**	CS	Blocks	3	4.80	n.e.
		Trt	3	6.20	0.0010
		Week	6	2.55	0.0293
		Trt*Week	18	0.23	0.9995
**Stomatal conductance**	CS	Blocks	3	30.04	n.e.
		Trt	3	6.67	0.0006
		Week	6	7.71	<.0001
		Trt*Week	18	0.71	0.7887
**Chlorophyll content**	CS	Blocks	3	28.47	n.e.
		Trt	3	27.37	<.0001
		Week	6	27.89	<.0001
		Trt*Week	18	0.76	0.7363

n.e.- Not estimated;
